# IGFBP7 regulates cell proliferation and migration through JAK/STAT pathway in gastric cancer and is regulated by DNA and RNA methylation

**DOI:** 10.1111/jcmm.70080

**Published:** 2024-10-01

**Authors:** Weilie Mo, Lijian Deng, Yun Cheng, Sen Ge, Jin Wang

**Affiliations:** ^1^ Department of General Surgery Changzhou No.7 People's Hospital Changzhou China; ^2^ Department of General Surgery Changzhou Geriatric Hospital affiliated to Soochow University Changzhou China; ^3^ Department of Oncology Changzhou No.7 People's Hospital Changzhou China; ^4^ Department of Oncology Changzhou Geriatric Hospital affiliated to Soochow University Changzhou China; ^5^ School of Public Health Suzhou Medical College of Soochow University Suzhou China

**Keywords:** drug sensitivity, gastric cancer, IGFBP7, JAK/STAT pathway, methylation

## Abstract

New biomarkers for early diagnosis of gastric cancer (GC), the second leading cause of cancer‐related death, are urgently needed. *IGFBP7*, known to play various roles in multiple tumours, is complexly regulated across diverse cancer types, as evidenced by our pancancer analysis. Bioinformatics analysis revealed that *IGFBP7* expression was related to patient prognosis, tumour clinicopathological characteristics, tumour stemness, microsatellite instability and immune cell infiltration, as well as the expression of oncogenes and immune checkpoints. GSEA links *IGFBP7* to several cancer‐related pathways. *IGFBP7* deficiency inhibited GC cell proliferation and migration in vitro. Furthermore, an in vivo nude mouse model revealed that *IGFBP7* downregulation suppressed the tumorigenesis of GC cells. Western blotting analysis showed that the JAK1/2‐specific inhibitor ruxolitinib could rescue alterations induced by *IGFBP7* overexpression in GC cells. Additionally, our bioinformatics analysis and in vitro assays suggested that *IGFBP7* is regulated by DNA methylation at the genetic level and that the RNA m^6^A demethylase FTO modulates it at the posttranscriptional level. This study emphasizes *the* clinical relevance of IGFBP7 in GC and its influence on cell proliferation and migration via the JAK/STAT signalling pathway. This study also highlights the regulation of *IGFBP7* in GC by DNA and m^6^A RNA methylation.

## 1 INTRODUCTION

Gastric cancer (GC) is the fifth most common cancer in the world and the second leading cause of cancer‐related death.^1^ Approximately 456,000 new cases of GC are diagnosed in China each year, and the mortality rates for men and women are 13.6% and 15.1%, respectively.^2^ Even in the United States, where medical technology has developed, GC has a high mortality rate.^3^ Patients with early GC can achieve good recovery after surgery; however, most of them are already in the middle or late stages when they are diagnosed and have lost the opportunity for surgery.^4^ Although various biomarkers, such as carcinoembryonic antigen (CEA) and carbohydrate antigen 724 (CA‐724), have been used in the clinical diagnosis of GC, their sensitivity, specificity, and reliability are still controversial.^5^, ^6^, ^7^ Therefore, it is important to find new biomarkers to diagnose early GC and improve patient prognosis.

Insulin‐like growth factor binding protein 7 (IGFBP7) is the first discovered IGFBP‐related protein of the IGFBP superfamily and is a secreted protein.^8^ As an important binding protein of insulin‐like growth factor, IGFBP7 is widely expressed in the brain, lungs, heart, gastrointestinal tract, pancreas, liver, prostate and so on. In addition, it can also be detected in serum, urine, cerebrospinal fluid, and amniotic fluid.^9^, ^10^ IGFBP7 has a variety of biological functions, including high‐efficiency collection of insulin to induce insulin resistance, promotion of endothelial angiogenesis, and regulation of cell proliferation, migration, apoptosis, and epithelial–mesenchymal transition (EMT).^11^, ^12^ IGFBP7 has been reported to play different roles in various tumours. For instance, IGFBP7 promoted colon cancer development through paracrine tumour‐stroma interactions.^13^ Low expression of IGFBP7 may be a good independent prognostic indicator for breast cancer.^11^ However, other studies have shown that IGFBP7 plays an adverse role in some tumours. Guo et al. reported that increased expression of IGFBP7 can inhibit the proliferation and invasion of HeLa cells in cervical cancer.^14^ IGFBP7 was found to be a p53 target gene inactivated in human lung cancer by DNA hypermethylation.^15^ Currently, the critical roles of IGFBP7 in GC remain largely controversial. High expression of *IGFBP7* promotes GC progression and is significantly associated with poor prognosis.^12^, ^16^ However, Kim et al. reported that IGFBP7 functions as a tumour suppressor in GC through an epigenetic pathway.^17^ A study by Liu et al. suggested that *IGFBP7* was downregulated in GC and that low expression of *IGFBP7* was associated with poor prognosis.^18^

Here, we aimed to explore the clinical significance and regulatory mechanism of IGFBP7 in GC via bioinformatics analysis and in vitro and in vivo experiments.

## 2 RESULTS

### 2.1 High expression of IGFBP7 has important clinical significance in GC

**FIGURE 1:**
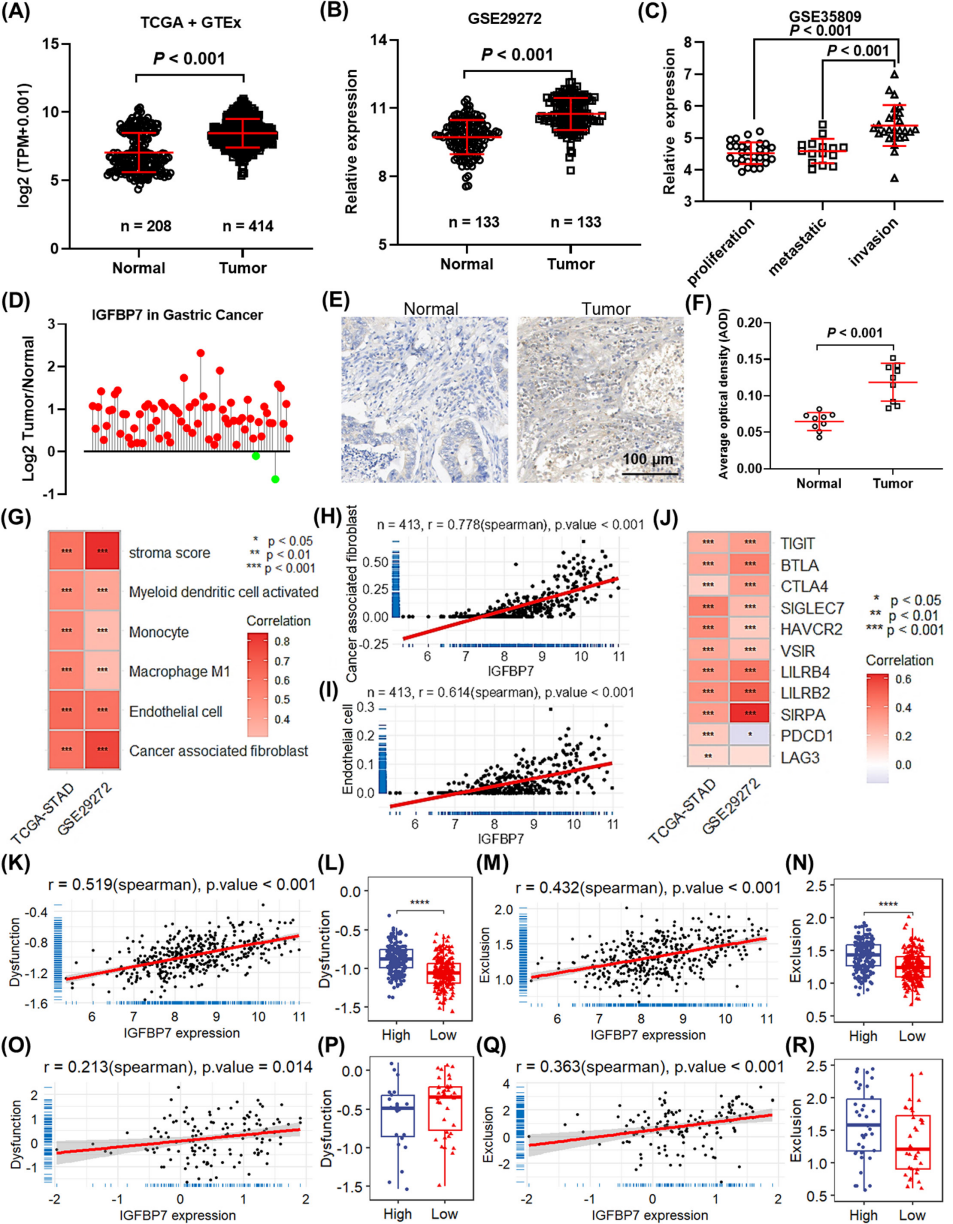
*IGFBP7* is highly expressed in gastric cancer tissues and is correlated with the immune response. (A) Differential expression of *IGFBP7* in tumour and normal tissues based on the TCGA and GTEx databases. (B) Differential expression of *IGFBP7* in tumour and normal tissues in the GSE29272 dataset. (C) Expression of *IGFBP7* in different types of tumour tissues based on the GSE35809 dataset. (D) Lollipop plot showing the log2‐transformed ratio of the expression between tumour and adjacent normal tissues in gastric cancer in the CPTAC dataset. Immunohistochemical staining analysis of IGFBP7 (E) and quantitative results (F) in clinical gastric cancer samples. (G) Heatmap showing the intersection of correlated immune cells with a correlation coefficient >0.3 in both the TCGA‐STAD and GSE29272 datasets. Scatter plots showing the correlation between *IGFBP7* expression and the infiltration of cancer‐associated fibroblasts (H) and endothelial cells (I) in the TCGA‐STAD cohort. (J) Heatmap showing the correlation between the expression of *IGFBP7* and immune checkpoint molecules in the TCGA‐STAD and GSE29272 datasets. Scatter plots showing the correlation between *IGFBP7* expression and T‐cell dysfunction (K) and exclusion (M) based on the TCGA‐STAD dataset. Box plots showing the different levels of T‐cell dysfunction (L) and exclusion (N) in the samples with high and low expression of *IGFBP7*. Scatter plots showing the correlation between *IGFBP7* expression and T‐cell dysfunction (O) and T‐cell exclusion (Q) based on the GSE29272 dataset. Box plots showing the different levels of T‐cell dysfunction (P) and exclusion (R) in the samples with high and low expression of *IGFBP7*.In the heatmap, * *p* < 0.05 for correlation analysis, ** *p* <0.01 for correlation analysis , *** *p* <0.001 for correlation analysis. In the box plots,* represents P < 0.05 between low and high groups. *** represents P < 0.05 between low and high groups. TCGA, The Cancer Genome Atlas; STAD, stomach adenocarcinoma. TIDE, tumour immune dysfunction and exclusion. GTEx, genotype‐tissue expression.

Based on the pancancer expression analysis, *IGFBP7* exhibited significant tumour‐type specificity (Figure S1A). In STAD, *IGFBP7* was upregulated in tumour tissues compared with normal tissues when only the TCGA database was used (Figure S1B) or when the TCGA and GTEx databases were combined (Figure 1A). In addition, *IGFBP7* was also highly expressed in tumours in another STAD dataset, GSE29272 (Figure 1B). Paired Student's *t*‐test also suggested *that IGFBP7* was significantly upregulated in STAD tumour tissues compared with paired normal tissues (Figure S1C,D). When samples were grouped based on tumour stage, *IGFBP7* was upregulated in stage II/III/IV tumours compared with stage I tumours (Figure S1E). With respect to tumour invasion depth, *IGFBP7* expression was significantly elevated in tumours with advanced invasion depth (Figure S1F). When considering the STAD grade, *IGFBP7* expression was greater in Grade 3 tumours than Grade 2 tumours (Figure S1G). In another STAD dataset, GSE35809, *IGFBP7* was found to be overexpressed in invasive tumours compared with in proliferative and metastatic tumours (Figure 1C). At the protein level, high IGFBP7 expression was also found in GC patients in the CPTAC GC dataset (Figure 1D). Additionally, immunohistochemical analysis of clinical GC samples also revealed significant upregulation of IGFBP7 in tumour tissues (Figure 1E,F). Survival analysis based on the TCGA database demonstrated that STAD patients with low *IGFBP7* expression levels had better OS, DFI and PFI (Figure S2A–C). Additionally, survival analysis based on the KM plotter online tool demonstrated that *IGFBP7* contributes to worse OS, PPS and FP in patients with STAD (Figure S2D–F). Importantly, *IGFBP7* expression was significantly negatively correlated with tumour TMB (Figure S1H) and stemness (Figure S1I). When considering the different transcripts produced by variable splicing, 2 protein‐coding transcripts, viz. Both ENST000000295666 and ENST00000514062 were upregulated in tumour tissues (Figure S3A,B) and contributed to poor prognosis (Figure S3C–E). Furthermore, these analyses indicated that *IGFBP7* may have important implications for the progression and prognosis of STAD.

To further identify the clinical significance of *IGFBP7*, the correlation between *IGFBP7* expression and immune cell infiltration was analysed. The results revealed that *IGFBP7* expression was significantly correlated with the infiltration of multiple immune cell types in both the TCGA‐STAD (Figure S4A) and GSE29272 (Figure S4B) datasets according to the XCELL algorithm. At the intersection of both datasets, the stroma score and the infiltration of five types of immune cells were found to be positively correlated with *IGFBP7* expression (Figure 1G). Among these immune cells, the infiltration of cancer‐associated fibroblasts and endothelial cells was most significantly correlated with *IGFBP7* expression in both the TCGA‐STAD (Figure 1H,I) and GSE29272 (Figure S4C,D) datasets. In addition, similar results were revealed based on the MCPCounter (Figure S4E) and EPIC (Figure S4F) algorithms. In addition, the correlation analysis suggested that *IGFBP7* was positively correlated with multiple checkpoint molecules (Figure 1J). Correlation analysis also revealed that the expression of *IGFBP7* was positively correlated with the expression of multiple immune regulatory genes (Figure S5A), including *ENTPD1*, *TGFB1*, *TGFBR1* and *CXCR4* (Figure S5B–E). Moreover, the significant correlation between *IGFBP7* expression and cancer‐associated fibroblasts was also verified by the TIDE online tool in both the TCGA‐STAD (Figure S4G) and GSE29272 (Figure S4H) datasets. Additionally, the T‐cell dysfunction and exclusion scores were found to correlate with *IGFBP7* expression and were markedly greater in samples with high *IGFBP7* expression in both the TCGA‐STAD (Figure 1K–N) and GSE29272 (Figure 1O–R) datasets. These results suggested that *IGFBP7* may contribute to the progression, immune cell infiltration and immune response of GC.

### 2.2 IGFBP7 serves as an oncogene in GC

**FIGURE 2:**
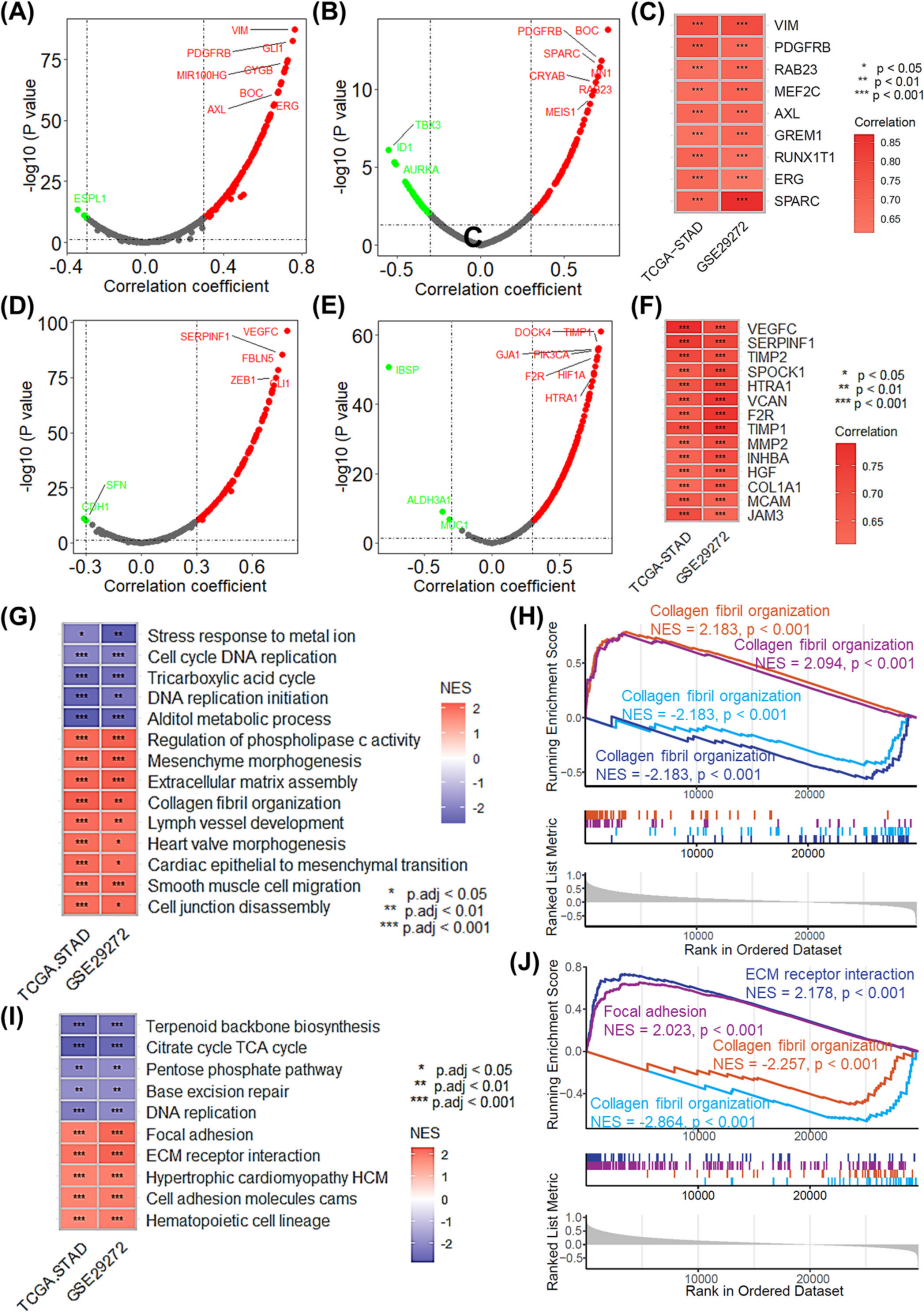
*IGFBP7* serves as an oncogene in GC. Volcano plots showing the correlation between the expression of *IGFBP7* and oncogenes based on the TCGA‐STAD (A) and GSE29272 (B) datasets. (C) Heatmap showing the intersection of correlated oncogenes with a correlation coefficient >0.6 in both the TCGA‐STAD and GSE29272 datasets. Volcano plots showing the correlation between the expression of *IGFBP7* and metastasis‐associated genes based on the TCGA‐STAD (D) and GSE29272 (E) datasets. (F) Heatmap showing the intersection of correlated metastasis‐associated genes with a correlation coefficient >0.6 in both the TCGA‐STAD and GSE29272 datasets. (G) Heatmap showing the results of GSEA of the biological processes associated with *IGFBP7* based on the TCGA‐STAD and GSE29272 datasets. (H) GSEA plots reveal that *IGFBP7* is correlated with several important biological processes, including collagen fibril organization, extracellular matrix assembly, cell cycle DNA replication and DNA replication initiation. (I) Heatmap showing the results of GSEA of the KEGG pathways associated with *IGFBP7* in the TCGA‐STAD and GSE29272 datasets. (J) GSEA plots reveal that *IGFBP7* is correlated with several important biological processes, including ECM receptor interaction, focal adhesion, DNA replication and the citrate cycle. In the heatmap, * *p* < 0.05 for correlation analysis, ** *p* <0.01 for correlation analysis , *** *p* <0.001 for correlation analysis. GSEA, gene set enrichment analysis; ECM, extracellular matrix; STAD, stomach adenocarcinoma; TCA, tricarboxylic acid cycle; TCGA: The Cancer Genome Atlas STAD, stomach adenocarcinoma.

According to the previous analysis, *IGFBP7* seems to be an oncogene. To evaluate the biological role of *IGFBP7* in GC, we conducted a correlation analysis of the expression of oncogenes and tumour metastasis‐related genes (MRGs). The results demonstrated that *IGFBP7* was positively correlated with multiple oncogenes in both the TCGA‐STAD (Figure 2A) and GSE29272 datasets (Figure 2B), including *VIM*, *PDGFRB*, *RAB23*, *MEF2C*, *AXL*, *GREM1*, *RUNX1T1*, *ERG* and *SPARC* (Figure 2C). Additionally, *IGFBP7* expression was correlated with the expression of tumour MRGs in both datasets (Figure 2D–F). Furthermore, gene set enrichment analysis (GSEA) was used to identify the potential biological processes and KEGG pathways related to *IGFBP7*. The results revealed that *IGFBP7* correlated with several vital cancer‐related biological processes (Figure 2G), including collagen fibril organization, extracellular matrix assembly, cell cycle DNA replication and DNA replication initiation (Figure 2H). In addition, *IGFBP7* is related to several cancer‐associated pathways (Figure 2I), including ECM receptor interaction, focal adhesion, DNA replication and the citrate cycle (Figure 2J). This analysis suggested that *IGFBP7* may serve as an oncogene in GC.

### 2.3 IGFBP7 knockdown inhibits GC cell proliferation and migration

**FIGURE 3:**
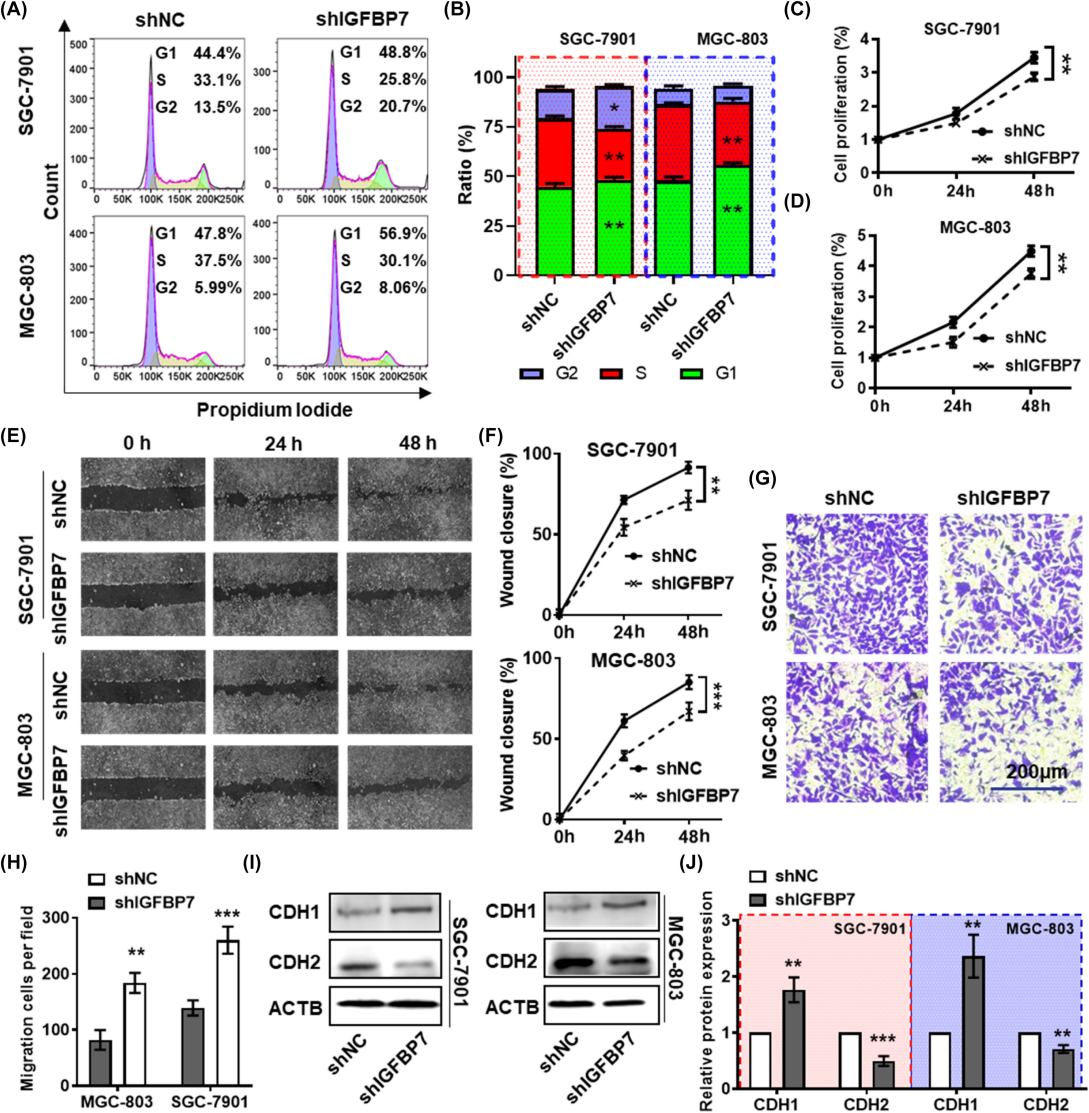
*IGFBP7* knockdown inhibits the proliferation and migration of GC cells. (A) PI staining was used to measure the cell cycle distribution of SGC‐7901 and MGC‐803 cells after *IGFBP7 knockdown*. (B) Statistical analysis of the cell cycle distribution of SGC‐7901 and MGC‐803 cells. A CCK‐8 assay was used to measure the proliferation of SGC‐7901 (C) and MGC‐803 (D) GC cells after *IGFBP7 knockdown*. (E) A wound healing assay and (F) statistical analysis were used to measure the cell migration ability of SGC‐7901 and MGC‐803 cells after *IGFBP7 knockdown*. (G) Transwell assays and (H) statistical analysis of the migration ability of SGC‐7901 and MGC‐803 cells after *IGFBP7 knockdown*. Western blot analysis (I) and statistical analysis (J) of CDH1 and CDH2 expression in *IGFBP7*‐knockdown cells. *, *p* < 0.05 between the two groups. **, p < 0.01 between the two groups. ***, p < 0.001 between the two groups. GC, gastric cancer. CDH1, E‐cadherin. CDH2, N‐cadherin.

To further validate the important functions of *IGFBP7* obtained from bioinformatics analysis, we knocked down *IGFBP7* by transfecting two GC cell lines with shRNA. Western blotting and ELISAs indicated that IGFBP7 was significantly deficient in the cell lysates and cell culture supernatants of shIGFBP7‐transfected MGC‐803 and SGC‐7901 cells (Figure S7A–C). To further validate the important functions of *IGFBP7* obtained from bioinformatics analysis, we knocked down *IGFBP7* by transfecting two GC cell lines with shRNA. Western blotting and ELISAs indicated that IGFBP7 was significantly deficient in the cell lysate and cell culture supernatant of shIGFBP7‐transfected MGC‐803 and SGC‐7901 cells (Figure S7A–C). In addition, cell cycle analysis demonstrated that the proportion of IGFBP7‐knockdown cells in the S phase was significantly lower than that of control cells, while the proportion of cells in the G1/G2 phase was greater (Figure 3A,B). The CCK‐8 assay revealed that IGFBP7 knockdown significantly inhibited the proliferation of MGC‐803 and SGC‐7901 cells (Figure 3C,D). In addition, cell scratch wound healing and transwell cell migration assays were used to evaluate the alteration of migration ability after the knockdown of *IGFBP7* in gastric cells. The results revealed that knocking down *IGFBP7* significantly attenuated the migration ability of both GC cell lines (Figure 3E–H). In addition, western blotting analysis revealed that the epithelial marker CDH1 was significantly upregulated, while the mesenchymal biomarker CDH2 was downregulated in cells with *IGFBP7* knockdown (Figure 3I,J).

### 2.4 IGFBP7 downregulation promotes erlotinib sensitivity in GC cells

**FIGURE 4:**
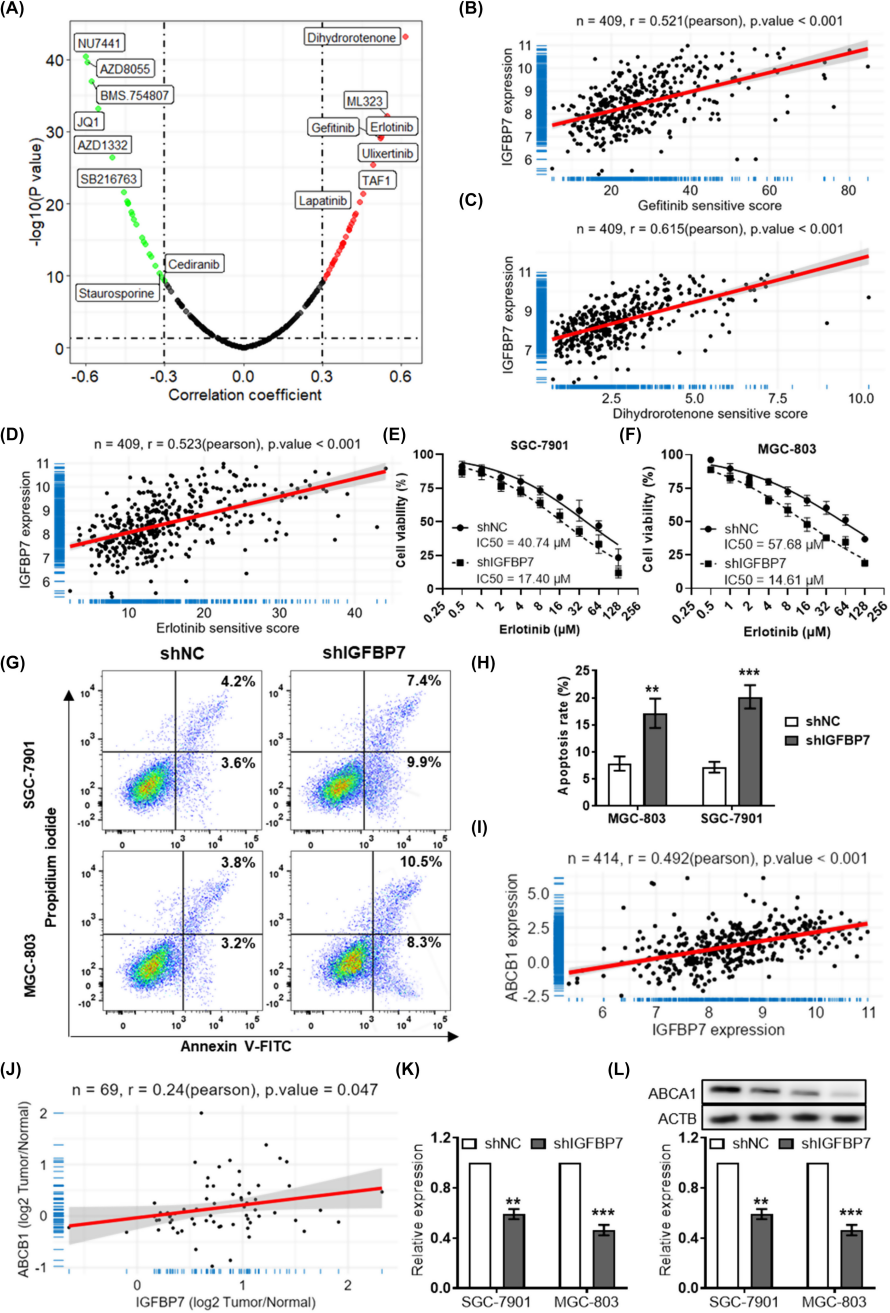
*IGFBP7* knockdown promotes erlotinib sensitivity in GC cells. (A) Volcano plot showing the results of the correlation analysis between *IGFBP7* expression and the sensitivity scores to multiple antitumor drugs. Scatter plots showing the correlation between IGFBP7 expression and the sensitivity scores to gefitinib (B), dihydrorotenone (C) and erlotinib (D) in the TCGA‐BLCA dataset. Dose–response curves of erlotinib treatment in *IGFBP7*‐knockdown SGC‐7901 (E) and MGC‐803 (F) cells. Representative images (G) and statistics (H) of the results of the PI/Annexin V‐FITC staining assay used to measure cell apoptosis in *IGFBP7*‐knockdown cells treated with erlotinib. (I) Scatter plot showing the correlation between the expression of *IGFBP7* and *ABCB1* in the TCGA‐BLCA dataset. (J) Scatter plot showing the correlation between the expression of IGFBP7 and ABCB1 in the gastric cancer dataset from the CPTAC database. (K) Relative expression of *ABCB1* in *IGFBP7*‐knockdown GC cells. (L) Western blotting showing the relative expression of ABCB1 in *IGFBP7*‐knockdown GC cells. **, *p* < 0.01 between the two groups. ***, *p* < 0.001 between the two groups. GC, gastric cancer. TCGA: The Cancer Genome Atlas STAD, stomach adenocarcinoma.

Correlation analysis revealed that the putative sensitivity scores of several antitumor drugs, including gefitinib (*r* = 0.521, Figure 4B), dihydrorotenone (*r* = 0.615, Figure 4C), and erlotinib (*r* = 0.532, Figure 4D), were correlated with IGFBP7 expression (Figure 4A). It should be noted that based on the principle of the OncoPredict package, the input sensitivity score is positively correlated with the IC50. The CCK‐8 assay indicated that cell viability was inhibited in *IGFBP7*‐knockdown cells treated with erlotinib (Figure 4E,F). The cell apoptosis rate was also increased in *IGFBP7*‐knockdown cells after treatment with erlotinib (Figure 4G,H). To preliminarily explore the mechanism by which IGFBP7 leads to erlotinib resistance in GC cells, we analysed two key molecules involved in drug resistance mechanisms, viz. *ABCB1* and *ABCG2*. Correlation analysis revealed that *IGFBP7* expression was significantly correlated with the expression of *ABCB1* (*r* = 0.496, Figure 4I) and ABCG2 (*r* = 0.219, Figure S6). Importantly, a significant correlation was also found at the protein level based on the CPTAC GC dataset (*r* = 0.257, Figure 4J). In addition, the mRNA and protein expression levels of ABCB1 were significantly reduced in two GC cell lines with *IGFBP7 knockdown* (Figure 4K,L).

### 2.5 IGFBP7 downregulation suppresses GC cell tumorigenesis in vivo

**FIGURE 5:**
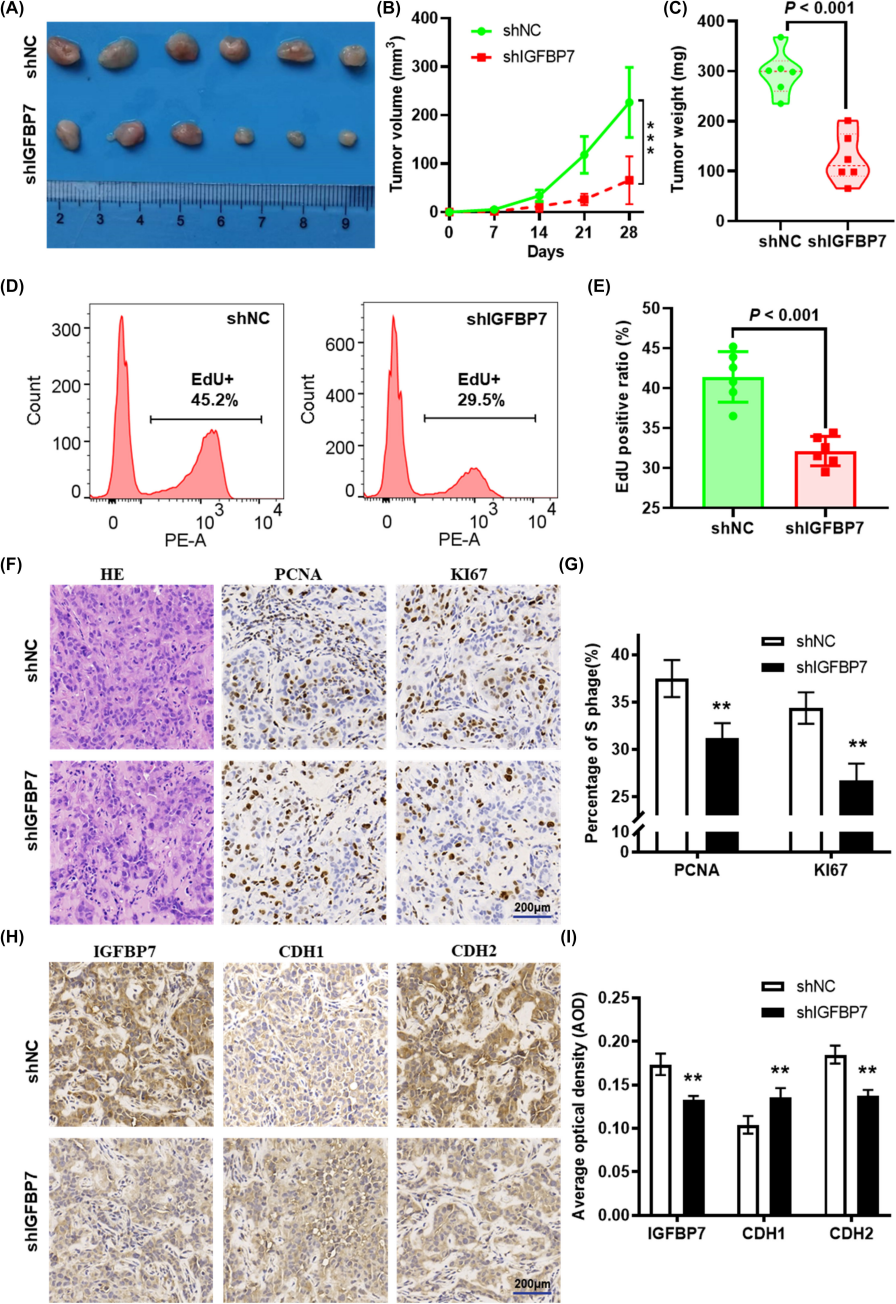
*IGFBP7* knockdown suppresses tumorigenesis in vivo. (A) Images of xenograft tumours formed in nude mice that were injected with *IGFBP7*‐knockdown cells or control cells. (B) Growth curve of tumours formed in mice. (C) The weight of the xenograft tumours isolated from nude mice. (D) Representative images and (E) quantified results of the EdU cell proliferation assay for single‐cell suspensions obtained from xenograft tumours. (F) Representative images of H&E staining and IHC staining of Ki67 and PCNA in xenograft tumours derived from nude mice. (G) The percentage of Ki67‐ and cyclin D1‐positive cells was quantified using ImageJ. (H) Representative images of H&E staining and IHC staining of IGFBP7, CDH1 and CDH2 in xenograft tumours derived from nude mice. (I) The average optical density of IGFBP7, CDH1 and CDH2 was quantified using the ImageJ plugin IHC profiler. Scale bar = 100 μm. **p < 0.01 vs. the blank group. IHC: Immunohistochemistry. CDH1, E‐cadherin. CDH2, N‐cadherin.

A xenograft animal model was used to further investigate the effects of *IGFBP7* on tumour growth in vivo. The results suggested that *IGFBP7* knockdown (shIGFBP7) significantly attenuated tumour growth compared with that in the control group (Figure 5A,B). Additionally, the weights of the tumours dissected from the mice in the shIGFBP7 group were significantly lower than those in the control group (Figure 5C). EdU assays revealed that the percentage of EdU‐positive cells in the tumour tissue of the shIGFBP7 group was significantly lower than that in the control group (Figure 5D,E). IHC staining revealed that the percentages of cells positive for two cell proliferation markers, viz. The levels of PCNA and Ki67 were notably reduced in the tumours dissected from the shIGFBP7 group (Figure 5F,G). In addition, the expression of CDH1 was amplified, while that of CDH2 was attenuated in the tumours dissected from the shIGFBP7 group (Figure 5H,I). Both in vitro and in vivo assays revealed that *IGFBP7* contributes to the proliferation and migration of gastric cells.

### 2.6 IGFBP7 promotes GC cell proliferation and migration through the JAK/STAT pathway

**FIGURE 6:**
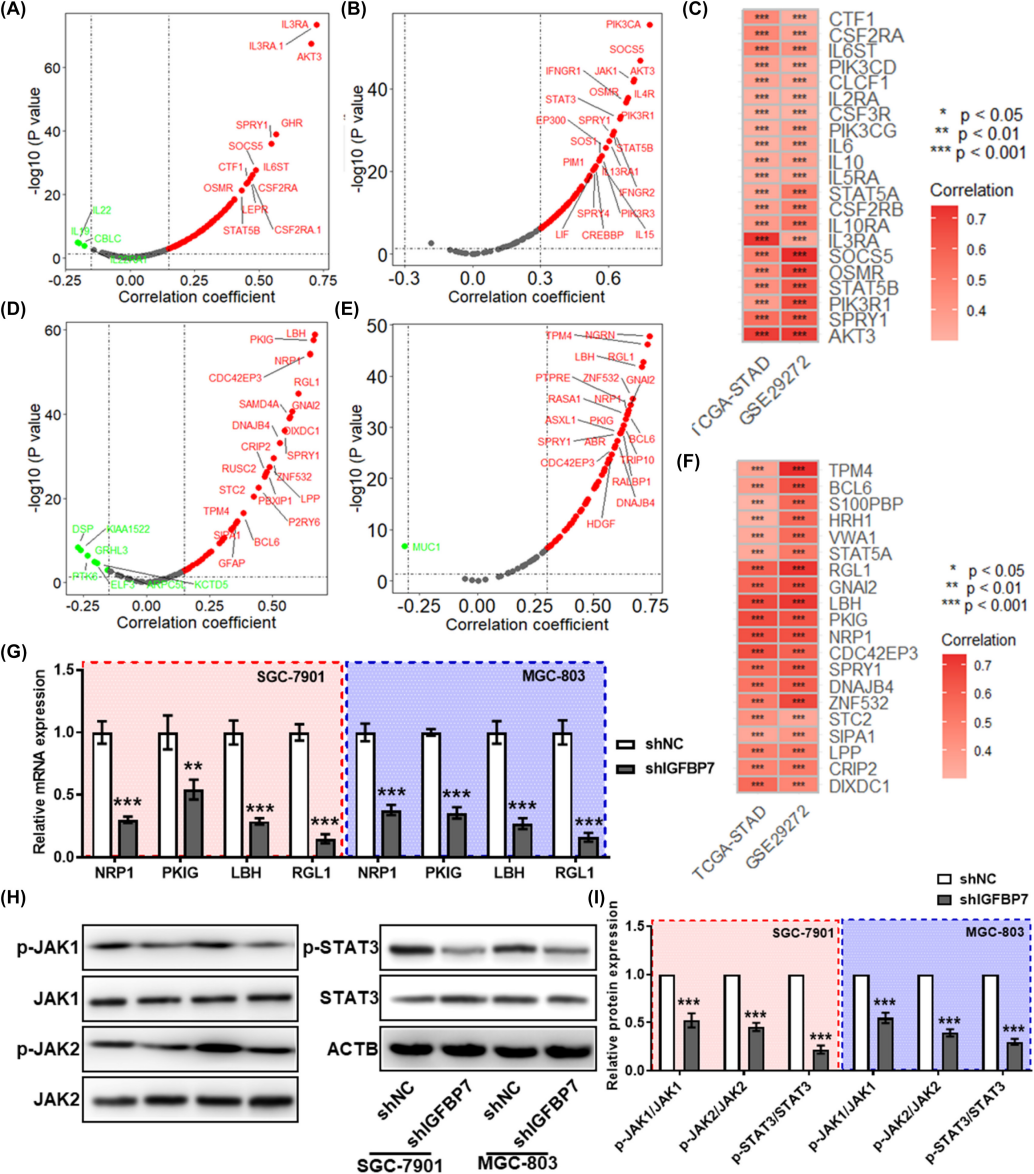
*IGFBP7* is speculated to regulate the JAK/STAT signalling pathway. Volcano plots showing the results of correlation analysis between the expression of *IGFBP7* and genes related to the JAK/STAT signalling pathway in the TCGA‐STAD (A) and GSE29272 (B) datasets. (C) Heatmap showing the results of the correlation analysis between the expression of IGFBP7 and genes related to the JAK/STAT signalling pathway in the TCGA‐STAD and GSE29272 datasets. Volcano plots showing the results of the correlation analysis between the expression of IGFBP7 and its target genes in the TCGA‐STAD (D) and GSE29272 (E) datasets. (F) Heatmap showing the results of the correlation analysis between the expression of IGFBP7 and its target genes in the TCGA‐STAD and GSE29272 datasets. (G) qPCR was used to analyse the changes in the expression of the top 8 predicted target genes of IGFBP7 in *IGFBP7*‐knockdown cells. (H–I) Western blotting was used to analyse the levels of phosphorylated and total JAK1/2 and STAT3 proteins in *IGFBP7*‐silenced cells. In the heatmap, * *p* < 0.05 for correlation analysis, ** *p* < 0.01 for correlation analysis , *** *p* < 0.001 for correlation analysis. In the bar plots, **, *p* < 0.01 between the two groups. ***, *p* < 0.001 between the two groups. TCGA, The Cancer Genome Atlas. STAD, stomach adenocarcinoma.

GSEA revealed that *IGFBP7* is related to the JAK/STAT signalling pathway, with an NES = 1.903 in the TCGA‐STAD dataset (Figure S8A). The genes involved in the JAK/STAT pathway were extracted from the KEGG website, and further correlation analysis revealed that *IGFBP7* was positively correlated with these genes in both the TCGA‐STAD and GSE29272 datasets (Figure 6A–C). In addition, a total of 99 high‐confidence target genes of STAT3 were predicted by using 5 online tools (Figure S8B), and they were significantly correlated with IGFBP7 in both the TCGA‐STAD and GSE29272 datasets (Figure 6D–F). qPCR revealed that the expression of the top eight predicted target genes was significantly downregulated in *IGFBP7*‐knockdown GC cells (Figure 6G). Western blotting analysis revealed that the protein levels of phosphorylated JAK1/2 and STAT3 were significantly reduced in *IGFBP7*‐knockdown GC cells (Figure 6H,I).

**FIGURE 7:**
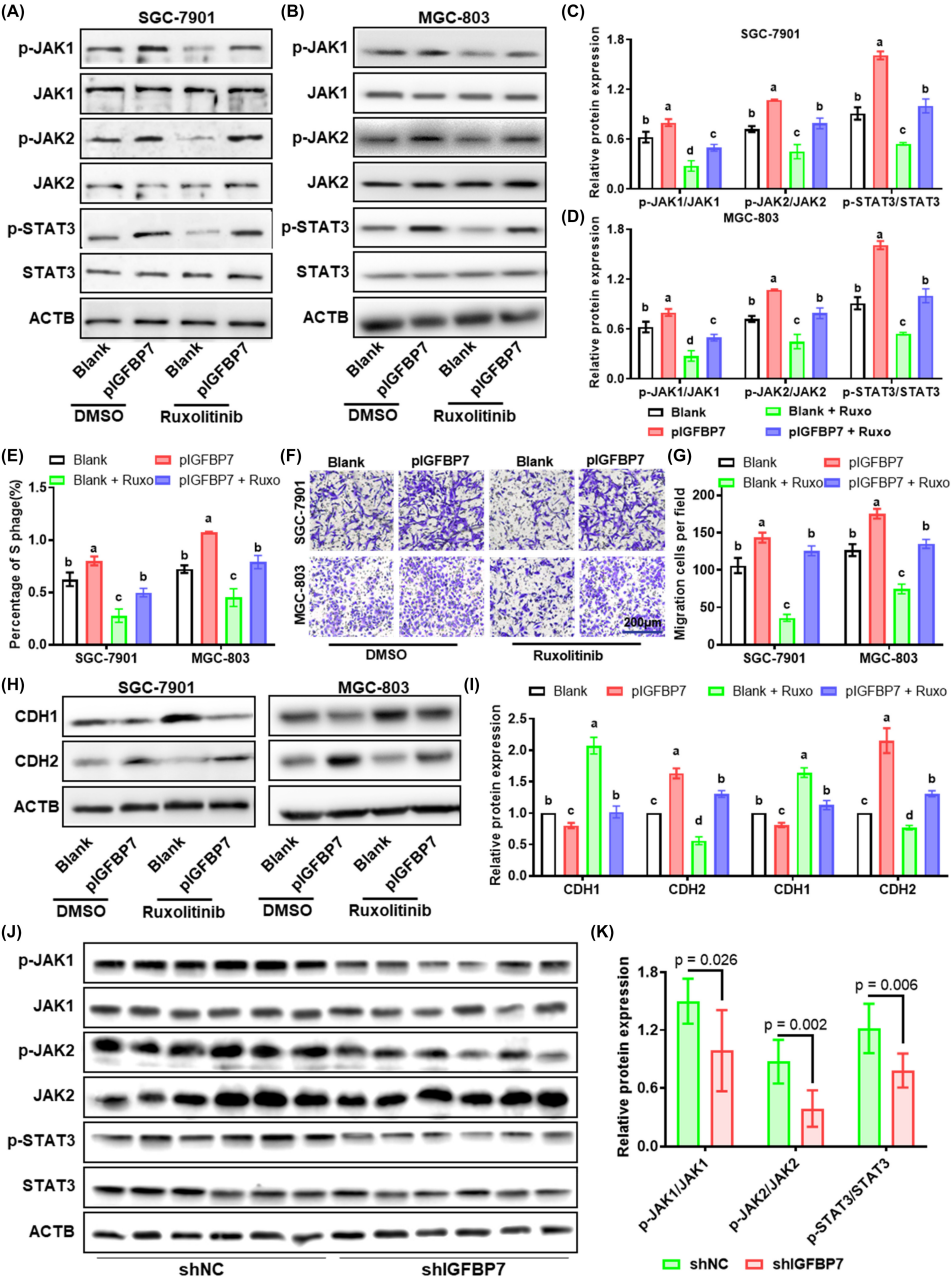
IGFBP7 promotes GC cell proliferation and migration through the JAK/STAT pathway. (A, B) Western blotting was used to analyse the levels of the phosphorylated and total JAK1/2 and STAT3 proteins in *IGFBP7*‐overexpressing and/or ruxolitinib (10 μM)‐treated GC cells, (C, D) and the quantitative results. (E) Statistical analysis of the percentage of S‐phase cells in *IGFBP7*‐overexpressing or/and ruxolitinib‐treated GC cells. Representative images (F) and statistics (G) of the results of the transwell migration assay in *IGFBP7*‐overexpressing and/or ruxolitinib‐treated GC cells. Western blotting analysis (H) and statistical analysis (I) of CDH1 and CDH2 expression in *IGFBP7*‐overexpressing and/or ruxolitinib‐treated GC cells. Groups sharing the same letter (e.g., “a”, “b”, “c”) indicate no significant difference between these groups. Western blot analysis (J) and statistical analysis (K) of the levels of the phosphorylated and total JAK1/2 and STAT3 proteins in xenograft tumours derived from nude mice. CDH1: E‐cadherin. CDH2: N‐cadherin. GC, gastric cancer.

To evaluate whether *IGFBP7* regulates the proliferation and migration of GC cells through the JAK/STAT signalling pathway, we treated cells with ruxolitinib (10 μM), a specific inhibitor of JAK1/2. *IGFBP7*‐overexpressing cells were generated by transfection with the *IGFBP7* plasmid, and the results revealed that IGFBP7 was upregulated in the cell culture supernatant (Figure S7D). Furthermore, the impact of IGFBP7 secreted by cells on the proliferation and migration ability of gastric cells was investigated through a Transwell coculture system (Figure S7E). Western blotting analysis revealed that the protein levels of phosphorylated JAK1/2 and STAT3 were significantly increased in GC cells cocultured with IGFBP7‐overexpressing cells, and ruxolitinib treatment significantly reversed this increase (Figure 7A–D). For cell proliferation and migration, cell cycle analysis and Transwell assays indicated that the percentage of GC cells in the S phase and the percentage of migrating cells were significantly increased in the group cocultured with *IGFBP7*‐overexpressing cells, while ruxolitinib treatment significantly reversed these increases (Figure 7E–G). At the molecular level, the epithelial marker CDH1 was significantly downregulated in the cells cocultured with *IGFBP7*‐overexpressing cells, while the mesenchymal marker CDH2 was significantly upregulated. In addition, ruxolitinib treatment significantly reversed the alterations in CDH1/2 expression induced by *IGFBP7* overexpression (Figure 7H,I). Moreover, the levels of phosphorylated JAK1/2 and STAT3 proteins were significantly lower in the tumours dissected from the shIGFBP7 group than in those from the shNC group (Figure 7J,K). These results demonstrated that increased IGFBP7 in GC cells can regulate proliferation and migration through the JAK/STAT signalling pathway.

### 2.7 IGFBP7 downregulation was regulated by DNA and mRNA methylation

**FIGURE 8:**
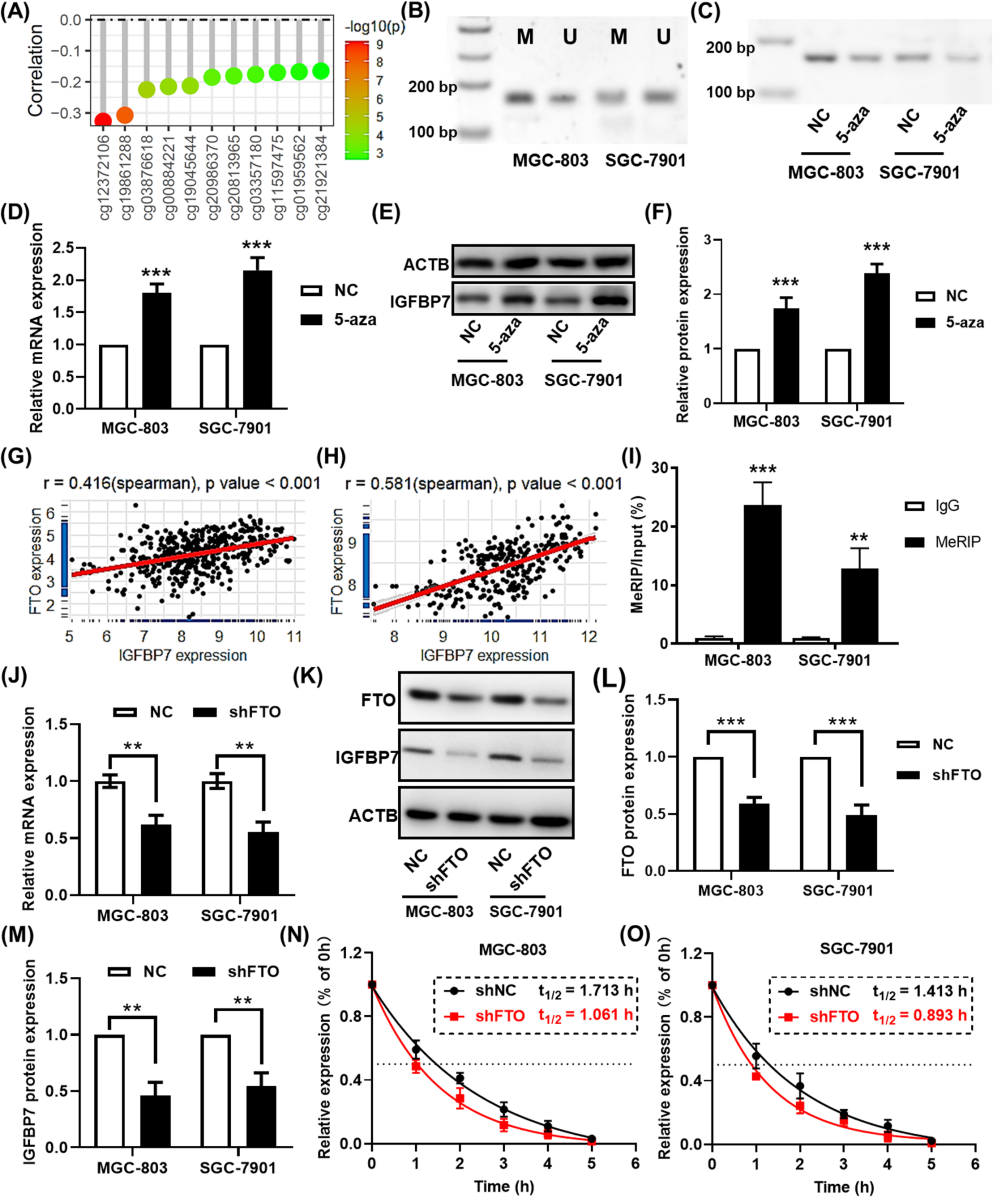
The expression of *IGFBP7* was modulated by DNA methylation at the gene level and m^6^A RNA methylation at the mRNA level. (A) Lollipop plot showing the results of the correlation analysis between IGFBP7 expression and the methylation of the probes targeting the promoter region of IGFBP7. (B) Methylation‐specific PCR analysis of the *IGFBP7* promoter in MGC‐803 and SGC‐7901 cells. (C) Methylation‐specific PCR analysis of the *IGFBP7* promoter in MGC‐803 and SGC‐7901 cells treated with 5‐aza‐2′‐deoxycytidine. (D) mRNA and (E, F) protein expression in MGC‐803 and SGC‐7901 cells treated with 5‐aza‐2′‐deoxycytidine. Scatter plots showing the correlation between the expression of *IGFBP7* and that of *FTO* in the TCGA‐STAD (G) and GSE29272 (H) datasets. (I) The m^6^A modification level of *IGFBP7* was measured by methylated RNA immunoprecipitation. (J) *IGFBP7* mRNA expression in GC cells was knocked down by FTO. (K–M) IGFBP7 and FTO protein expression in GC cells with FTO knockdown. (N, O) IGFBP7 mRNA decay curves in FTO‐knockdown cells after blocking transcription with actinomycin D treatment. **, *p* < 0.01 between the two groups. ***, *p* < 0.001 between the two groups. GC, gastric cancer; TCGA, The Cancer Genome Atlas STAD, stomach adenocarcinoma.

Analysis of the TCGA database revealed that the expression of *IGFBP7* was significantly negatively correlated with that of DNMT3B and DNMT1 (Figure S9A,B). In addition, further analysis revealed that the expression of *IGFBP7* was significantly negatively correlated with the methylation of the probes targeting the promoter region of *IGFBP7* (Figure 8A). Using the MethyPrimer online tool, a CpG island was found in the promoter region of *IGFBP7*, and a primer set for MSP was designed (Figure S9C). The MSP assay revealed methylation in the *IGFBP7* promoter region in GC cells (Figure 8B). After treating GC cells with the methylase inhibitor 5‐azacytidine (5‐aza), the methylation level was reduced compared with that in the DMSO control group (Figure 8C), while *IGFBP7* mRNA and protein expression were upregulated (Figure 8D–F). These results suggested that *IGFBP7* downregulation was modulated by DNA methylation at the genetic level.

Based on the correlation analysis, a significant positive correlation was found between the expression of *IGFBP7* and the m^6^A demethylase FTO in the TCGA‐STAD dataset (Table S1, Figure 8G). Additionally, the positive correlation between the expression of *FTO* and *IGFBP7* was verified in the GSE29272 dataset (Figure 8H). With the use of the SRAMP m^6^A site prediction tool, one putative m^6^A site with high confidence was identified (Figure S9E). Further meRIP qPCR analysis confirmed the m^6^A modification of this site in both gastric cell lines (Figure 8I). To determine the regulatory mechanism of FTO on *IGFBP7*, the level of FTO was reduced by shRNA transfection. qPCR analysis revealed that *IGFBP7* was downregulated after FTO was knocked down (Figure 8J). In addition, western blotting analysis showed consistent changes in protein levels in both gastric cell lines (Figure 8K–M). Furthermore, an RNA stability assay using actinomycin D revealed that FTO knockdown accelerated the degradation of *IGFBP7*, and the half‐life of *IGFBP7* mRNA decreased from 1.713 h to 1.061 h in MGC‐803 cells (Figure 8N) and decreased from 1.413 h to 0.893 h in SGC‐7901 cells (Figure 8O). These results suggested that *IGFBP7* was modulated by FTO and m^6^A modification at the mRNA level.

## 3 DISCUSSION

Due to the difficulty of early diagnosis of GC, many patients are already diagnosed at an advanced stage, resulting in a poor prognosis. The identification of more accurate molecular biomarkers and drug‐forming targets is urgently needed. *IGFBP7* may have tumour type‐specific effects on cancer and may even exhibit contradictory effects in some tumours. *IGFBP7* functions as a tumour suppressor gene in breast cancer, thyroid cancer and melanoma by inducing cell apoptosis and cell senescence.^19^, ^20^, ^21^ In contrast, several other studies have suggested its oncogenic role in colon cancer and oesophageal squamous cell carcinoma.^13^, ^22^ Our pancancer analysis also revealed the complicated regulation of *IGFBP7* in different cancer types.

The generally poor prognosis of GC patients is usually due to difficulty in early diagnosis and drug resistance.^23^ Previous studies have indicated that *IGFBP7* contributes to the poor prognosis of GC patients,^12^ which is consistent with our current findings. In addition, our results revealed that *IGFBP7* was correlated with the sensitivity to several antitumor drugs. The EGFR family is one of the most studied receptor protein tyrosine kinases and antitumor drug targets due to its general role in signal transduction and tumorigenesis.^24^ Our results suggested that the sensitivity of GC cells to several EGFR inhibitors, including erlotinib, gefitinib and lapatinib, is closely related to *IGFBP7* expression and that knocking down *IGFBP7* significantly increased erlotinib sensitivity in GC cells. One of the most compelling and effective aspects in the study of tumour resistance mechanisms is the investigation and modulation of the drug efflux pumps embedded in the cell membrane (ABC transporters) that confer resistance.^25^ To date, many members of the ABC family have been found to be overexpressed in cancer cells and are responsible for transporting certain antitumor drugs out of the cell, thereby preventing the excessive accumulation of drugs within the cell or blocking pathways essential for cell survival.^25^, ^26^ The majority of studies on drug efflux ABC proteins, particularly ABCB1 and ABCG2, which are the main ABC transporters frequently overexpressed in multidrug‐resistant (MDR) cancer cells and capable of expelling a variety of xenobiotic substances, have focused on their inhibitors.^27^, ^28^ These inhibitors can attenuate their ability to expel antitumor drugs. Our findings revealed a correlation between ABCB1 expression and IGFBP7, suggesting a potential molecular mechanism by which IGFBP7 regulates resistance to erlotinib.

Recently, the tumour microenvironment (TME) has attracted increasing attention due to its important role in tumour immunosuppression, distant metastasis and drug resistance.^29^ The TME is mainly composed of tumour cells, infiltrating immune cells, cancer‐related stromal cells, endothelial cells and other components.^30^, ^31^ As one of the most important stromal cells in the TME, cancer‐related fibroblasts (CAFs) typically exhibit cancer‐promoting effects and can participate in multiple stages of tumour development through different pathways.^32^, ^33^ Recent research has shown that IGFBP7 promotes the polarization of tumour‐associated macrophages (TAMs) via the FGF2/FGFR1/PI3K/AKT axis, thereby facilitating the progression of GC.^34^ Our analysis revealed that *IGFBP7* expression was positively correlated with the stroma and microenvironment scores, as well as with the infiltration scores of endothelial cells and CAFs, revealing that IGFBP7 may contribute to the infiltration of several types of protumor cells. In addition, *IGFBP7* was correlated with the expression of several immune checkpoint molecules. Under physiological conditions, immune checkpoint genes can regulate the immune system by stimulating or suppressing immune responses, and this regulatory mechanism is common in tumours.^35^ These results suggest that *IGFBP7* may serve as a potential biomarker for the prediction of prognosis, immune microenvironment homeostasis, and the response to immune checkpoint blockade therapy.

There is a lack of studies on the biological functions of *IGFBP7* in cancer, especially in GC. In breast cancer, the overexpression of *IGFBP7* could contribute to the inhibition of cell proliferation and migration by inducing cell senescence and apoptosis pathways.^20^ In contrast, IGFPB7 can promote anchoring growth in malignant mesenchymal cells and epithelial cells with an EMT phenotype in colon cancer.^13^ In this study, *IGFBP7* was found to be positively correlated with multiple oncogenes, mainly cell migration‐ and proliferation‐related genes. Additionally, GSEA revealed that *IGFBP7* was related to several cancer‐related pathways, including cell adhesion molecule, extracellular matrix receptor interaction, cell cycle, DNA replication and base excision repair pathways. Further in vitro and in vivo assays also revealed that *IGFBP7* deficiency could attenuate the proliferation and migration of GC cells. Mechanistically, the JAK1/2 inhibitor ruxolitinib significantly rescued the increase in cell proliferation and migration caused by the overexpression of *IGFBP7*, indicating that the JAK–STAT signalling pathway may mediate the procancer effect of IGFBP7. The JAK/STAT signalling pathway is considered one of the central communication nodes in cellular functions, and its dysregulation has been confirmed to be associated with various cancers, inflammation, and autoimmune diseases.^36^ Recent studies have revealed that the IL‐6/JAK2/STAT3 signalling pathway is aberrantly activated in cells resistant to erlotinib and osimertinib,^37^ further elucidating the mechanism by which IGFBP7 regulates resistance to erlotinib.

*IGFBP7* was reported to be regulated by promoter DNA methylation in multiple cancer types, including prostate cancer,^38^ colon cancer,^39^ oesophageal adenocarcinoma^40^ and non‐small cell lung cancer.^41^ Our bioinformatics analysis and in vitro assays revealed that *IGFBP7* upregulation in GC was modulated by DNA methylation at the genetic level. N6‐methyladenosine (m^6^A) is the most common and conserved modification of eukaryotic RNA and plays a crucial role in both physiological and pathological conditions, especially in the occurrence and development of different types of cancer.^42^ As the first discovered RNA m^6^A demethylase, FTO is often dysregulated and plays important roles in various types of cancer.^43^ In this study, *IGFBP7* was modulated by m^6^A modification and FTO at the posttranscriptional level. These results suggested that *IGFBP7* is a potential regulator of cell proliferation and migration in GC and is regulated by DNA methylation at the genetic level and m6A RNA methylation at the posttranscriptional level.

In conclusion, this study indicated that *IGFBP7* has important clinical significance in GC and is associated with immune cell infiltration, such as that of cancer‐related fibroblasts, and the sensitivity to antitumor drugs, such as erlotinib. Functionally, knocking down *IGFBP7* attenuates the proliferation and migration of GC cells. Mechanistically, the JAK/STAT signalling pathway mediates the procancer effect of IGFBP7, and the upregulation of *IGFBP7* in GC is regulated by DNA methylation and m^6^A RNA methylation.

## 4 MATERIALS AND METHODS

### 4.1 Datasets

The integrated transcripts per million (TPM)‐normalized TCGA‐GTEx dataset, methylation data and clinicopathological data of patients with STAD were obtained from the Xena browser (https://xenabrowser.net/datapages/?cohort=TCGA%20TARGET%20GTEx).^44^ Two Gene Expression Omnibus (GEO) datasets, viz. The GSE35805^45^ and GSE29272^46^ datasets were downloaded from the GEO database (https://www.ncbi.nlm.nih.gov/geo/query/acc.cgi). The gene expression data were grouped and analysed according to tumour stage, invasion depth and tumour grade.

### 4.2 Clinical samples

In this study, we collected tumour tissues and adjacent normal tissues from 6 GC patients at the Seventh People's Hospital of Changzhou City. All participating patients were fully informed about the purpose, process, and potential risks of the study and provided their voluntary participation through the signing of informed consent forms. This study strictly adhered to relevant ethical guidelines and regulations and received approval from the Ethics Committee of the Seventh People's Hospital of Changzhou City (Approval No.: EC2023‐0415), ensuring the ethical and legal integrity of the research. The collection, processing, and analysis of samples were carried out in accordance with internationally recognized ethical standards and procedures aimed at maximizing the protection of patient rights.

### 4.3 Survival analysis

Correlations between the expression of the *IGFBP7* gene and first progression (FP), overall survival (OS) and postprogression survival (PPS) in GC patients were analysed by using the Kaplan–Meier plotter online tool (http://kmplot.com/analysis/).^47^ Additionally, the stomach adenocarcinoma (STAD) dataset was used to analyse the prognostic value of *IGFBP7* in GC patients. In all analyses, patients were divided into high‐ and low‐expression groups based on the median expression of *IGFBP7*. The “survminer” package (https://cran.r‐project.org/web/packages/survminer/index.html) of the R program was used for survival analysis and plotting of Kaplan–Meier curves.

### 4.4 Correlation analysis

To further explore the biological role and clinical significance of *IGFBP7*, correlation analysis was performed between *IGFBP7* and oncogene expression, tumour mutation burden (TMB), tumour stemness, microsatellite instability (MSI), immune regulatory gene expression, and immune cell infiltration. Correlation analysis was performed with the Spearman method based on the “psych” package.

The oncogenes were extracted from the ONGene database (http://www.ongene.bioinfo‐minzhao.org).^48^ HCMDB (http://hcmdb.i‐sanger.com/index) is an integrated database that has annotated thousands of potentially MRGs.^49^

The Genomics of Drug Sensitivity in Cancer (GDSC) database was developed by the Sanger Research Institute to collect data on the sensitivity and response of tumour cells to drugs.^50^ “OncoPredict” was used to calculate the drug sensitivity of each sample in the training and validation datasets based on the GDSC V2.0 database.^51^ Due to the sensitivity score being calculated based on the IC50 and sequencing data of multiple cell lines as the training set, the score obtained was positively correlated with the IC50 and negatively correlated with drug sensitivity.

### 4.5 GSEA

To predict the potential biological processes related to *IGFBP7*, GSEA was performed based on the gene set constructed based on the correlation analysis. The molecular signature data are available from https://www.gsea‐msigdb.org/gsea/downloads.jsp. Both Gene Ontology (GO) biological process and Kyoto Encyclopedia of Genes and Genomes (KEGG) pathway analyses were performed.

### 4.6 shRNA and overexpression plasmid construction

*IGFBP7* shRNA sequences were designed according to BLOCK‐iT™ RNAi Designer (https://rnaidesigner.thermofisher.com/rnaiexpress), and the annealed double‐stranded shRNA was cloned and inserted into the pGreen vector. After testing the knockdown efficiency of several candidate shRNAs, the sequence 5’‐GGGTCACTATGGAGTTCAAAG‐3′ targeting *IGFBP7* and the sequence 5’‐GCAGCTGAAATATCCTAAACT‐3′ targeting FTO were selected for subsequent experiments. A scrambled nonspecific control shRNA (shNC) was also cloned and inserted into the same vector and used as a negative control. For overexpression, the full‐length coding sequence of *IGFBP7* was amplified and cloned and inserted into the pCDH plasmid.

### 4.7 Cell culture and transfection

The human GC cell lines SGC‐7901 and MGC‐803 were purchased from the American Type Culture Collection (ATCC). All cells were cultured in DMEM (Thermo Fisher Scientific, Inc.) supplemented with 10% FBS (Thermo Fischer Scientific, Inc.) at 37°C in the presence of 5% CO_2_.

GC cells were seeded in 6‐well plates in each well and grown for 24 h. Then, the cells were transfected with 2.5 μg of shIGFBP7 or shNC using Lipofectamine 6000 reagent (Beyotime, China) following the manufacturer's protocol.

### 4.8 Cell cycle analysis

The cells were initially adequately trypsinized and subsequently fixed with 70% ethanol and incubated at −20°C overnight. Then, the cells were washed twice with PBS and stained with 10 μg/mL RNase A and 50 μg/mL PI staining buffer (Beyotime Institute of Biotechnology, China) for 30 min. BD FACS Canto II and FlowJo V10.3 software (FlowJo LLC, Ashland, OR, USA) were used to analyse the cell cycle data.

### 4.9 Cell proliferation assays

Cells were seeded into 6‐well plates and incubated with 10 μM EdU for 2 h. Then, the cells were fixed using 4% paraformaldehyde and permeabilized with 0.3% Triton X‐100 in PBS. Subsequently, the cells were subjected to incubation with a click reaction solution (Beyotime Institute of Biotechnology, China). Within 24 h, images were captured using an inverted fluorescence microscope, and the data were analysed utilizing NIH ImageJ software (version 1.8.0).

### 4.10 Cell migration assays

For the wound healing assay, first, cells were added to 6‐well plates. When the confluence of the cells exceeded 90%, they were cultured in serum‐free culture medium (DMEM). Subsequently, artificial wounds were made with a 100 mL pipette tip. After 24 h, the wound closure distance was recorded by using an inverted microscope.

### 4.11 Cell invasion assays

MGC‐803 and SGC‐7901 cells in each group were plated onto a Matrigel‐coated membrane in the upper well of each Transwell membrane (Corning, Inc., USA), and 1 mL of medium without FBS and 2 mL of complete medium were added to the bottom chamber. After 24 h of incubation at 37°C with 5% CO_2_, the cells were fixed in methanol and stained with 0.5% crystal violet for 30 min. Finally, phosphate‐buffered saline (PBS, Gibco, USA) was used to wash the cells in the upper chamber three times. The cells were imaged using a microscope and then assessed with NIH ImageJ software (version 1.8.0).

### 4.12 Xenograft model

Four‐ to six‐week‐old male specific‐pathogen‐free (SPF) BALB/c nude mice (Vitalriver, Inc., USA) were used for these in vivo xenotransplantation assays. The mouse care and experimental protocols used were approved by the Institutional Animal Care and Use Committee of Soochow University. SGC‐7901 cells stably transfected with shNC or shIGFBP7 were individually resuspended in 100 μL of PBS (5 × 10^7^ cells/ml) and then injected subcutaneously into the nude mice. Tumour volumes and weights were measured every week. In addition, the collected tissues were separated into two sections, one of which was fixed with 4% paraformaldehyde and subjected to immunohistochemistry (IHC), while the other part was stored at −80°C for qRT–qPCR or Western blotting analysis. All mice were euthanized at 28 days after inoculation.

### 4.13 Immunohistochemistry

After deparaffinization and hydration for staining, the tissue sections were incubated with specific primary antibodies overnight. The primary antibodies used for IHC were as follows: Ki67 (1:100, ProteinTech Group, Inc., USA), PCNA (1:100, ProteinTech Group, Inc., USA), CDH1 (1:100, ProteinTech Group, Inc., USA) and CDH2 (1:100, ProteinTech Group, Inc., USA). Then, the sections were incubated with a goat anti‐rabbit secondary antibody for 20 min and with streptavidin‐HRP for 30 min at room temperature. After thorough washing, the sections were stained with haematoxylin. Images were obtained under a microscope, and the average optical density (AOD) was quantified using ImageJ software.

### 4.14 DNA methylation analysis

To evaluate the potential role of DNA methylation in the regulation of *IGFBP7*, the correlations between *IGFBP7* and DNA methylase‐encoding genes, including *DNMT1*, *DNMT3A* and *DNMT3B*, were analysed. Additionally, correlations between the values of the methylation probes within the *IGFBP7* gene and *IGFBP7* expression were analysed. To detect the methylation of the promoter of GC cells, the MethyPrimer online tool (http://www.urogene.org/methprimer/) was used to design the primer pairs used for methylation‐specific PCR (MSP).^52^

For MSP, a DNA Purification Kit was used to extract genomic DNA from cells. According to the manufacturer's protocol, an Epitect Bisulfite kit was used to modify the genomic DNA with sodium bisulfite. AmpliTaq Gold was used for the MSP assays, and the products were subsequently separated on 2% agarose gels containing GelRed® nucleic acid gel stain. The IGFBP7 promoter region of each modified DNA sample was amplified to confirm that the DNA was successfully modified with bisulfite. The primers synthesized by Genewiz (China) used in these assays were as follows: Site1_MF, 5′‐ AAATTAGAGGGTGGAAGAGTCGT‐3′; site1_MR, 5′‐ CTACTAACGTCGAAAAATAAACGAA‐3′; site1_UF, 5′‐ AGAAATTAGAGGGTGGAAGAGTTGT‐3′; and site1_UR, 5′‐ CTACTAACATCAAAAAATAAACAAA‐3′. Additionally, cells treated with 20 μM 5‐aza‐2′‐deoxycytidine (5‐aza, a methyltransferase inhibitor) were used to verify the DNA methylation regulation of *IGFBP7*.

### 4.15 m6A RNA immunoprecipitation (meRIP) assay

The sequence‐based RNA adenosine methylation site predictor (SRAMP) online tool was used to predict m^6^A modifications in *IGFBP7*. Based on the predicted m6A primer pairs, we designed corresponding primers for meRIP‐PCR/qPCR. A Magna MeRIP m^6^A Kit (cat. no. 17–10,499, Millipore Sigma, MA, USA) was used to verify m^6^A modification in GC cells following the manufacturer's instructions. In brief, anti‐m^6^A antibody‐coated magnetic beads were coimmunoprecipitated with 150 μg of fragmented mRNA according to the protocols of the GenSeqTM m6A‐MeRIP Kit (GenSeq, Beijing, China). The eluent was used for m6A RNA elution and purification. PCR and reverse transcription (RT)‐qPCR assays were then performed on the abundance of eluted RNA samples with gene‐specific primers to analyse the mRNA levels of m^6^A sites.

### 4.16 Western blot

RIPA lysis buffer (Beyotime, China) was used to extract total protein from MGC‐803 and SGC‐7901 cells. Protein quantification was performed by using an Enhanced BCA Kit (Beyotime, China). Sodium dodecyl sulfate–polyacrylamide gel electrophoresis (SDS–PAGE) was used to separate equivalent amounts of proteins, and 30 μg of protein was then transferred onto a polyvinylidene difluoride (PVDF) membrane (Millipore Sigma, Billerica, MA). After being blocked in 5% BSA, the membranes were incubated with primary antibodies overnight at 4°C. The primary antibodies used were as follows: anti‐FTO (1:1000 dilution, ProteinTech Group, Inc., USA), anti‐IGFBP7 (1:1000 dilution, ProteinTech Group, Inc., USA), anti‐E‐cadherin (CDH1, 1:1000 dilution, ProteinTech Group, Inc., USA), anti‐N‐cadherin (CDH2, 1:1000 dilution, ProteinTech Group, Inc., USA), anti‐STAT3 (1:1000 dilution, ProteinTech Group, Inc., USA), anti‐JAK1 (1:5000 dilution, ProteinTech Group, Inc., USA), and anti‐JAK2 (1:1000 dilution, Cell Signalling Technology, Inc., USA). Anti‐GAPDH (1:1000 dilution, Cell Signalling Technology Inc., USA) was used as a loading control. The membranes were then incubated with HRP‐labelled secondary antibodies at room temperature for 2 h. The membranes were washed three times with TBST. The ECL substrate and the GeneTools GBox (Syngene) system were used to visualize the protein bands. The protein bands were scanned and quantified using ImageJ software (National Institutes of Health). GAPDH was used as the control.

### 4.17 RT–qPCR

According to the manufacturer's instructions, total RNA was extracted with TRIzol (Invitrogen, Thermo Fisher Scientific, Inc., Waltham, MA, USA). Subsequently, a RevertAid First Strand cDNA Synthesis Kit (Thermo Fisher Scientific, Inc., Waltham, MA, USA) was used to reverse transcribe total RNA into complementary DNA (cDNA) according to the manufacturer's protocol. qRT–PCR was performed on a QuantStudioTM 6 Flex RT–qPCR System (Applied Biosystems, Thermo Fisher Scientific, Inc., Foster City, CA, USA) with a FastStart Universal SYBR Green Master Mix (ROX) Kit (Roche Diagnostics, Switzerland). *GAPDH* was used as the internal control gene. The qPCR primer sequences for *IGFBP7* were as follows: IGFBP7_F 3’‐CGAGCAAGGTCCTTCCATAGT‐5′ and IGFBP7_R 3’‐GGTGTCGGGATTCCGATGAC‐5′.

### 4.18 RNA stability assay

Stably transfected shFTO and control GC cells were seeded into a 6‐well plate. The cells were collected at 0, 1, 2, 3, 4 and 5 h after treatment with actinomycin D (ACTD, final concentration, 10 μg/mL). Total RNA was isolated using TRIzol® reagent for qPCR to detect *IGFBP7* mRNA levels. The *IGFBP7* mRNA decay rate was determined using nonlinear regression curve fitting (one‐phase decay).

### 4.19 Statistical analysis

GraphPad V8.3.0 software (GraphPad Software, LLC) was used for the statistical analyses, and the data are presented as the means ± standard deviations. Student's t test and ANOVA (analysis of variance) were used to determine whether there was a statistically significant difference between the means of two or more groups, respectively. For all the statistical tests, *a P* value <0.05 was considered to indicate statistical significance.

## AUTHOR CONTRIBUTIONS

**Weilie Mo:** Conceptualization (equal); data curation (equal); funding acquisition (equal); methodology (equal); software (equal); writing – original draft (equal). **Lijian Deng:** Methodology (equal); validation (equal); writing – review and editing (equal). **Yun Cheng:** Validation (equal); writing – review and editing (equal). **Sen Ge:** Validation (equal); writing – review and editing (equal). **Jin Wang:** Conceptualization (equal); funding acquisition (equal); investigation (equal); methodology (lead); project administration (lead); resources (equal); software (equal).

## FUNDING INFORMATION

This study was funded by the Changzhou Applied Basic Research Program of the Key Research Project (project no. CJ20210098), the Top Talent of Changzhou “The 14th Five‐Year Plan”, the High‐Level Health Talents Training Project (project no. 2022CZBJ095) and the China Postdoctoral Science Foundation (project no. 2023 M732527).

## CONFLICT OF INTEREST STATEMENT

The authors declare no conflicts of interest.

## CONSENT

Not applicable.

## Supporting information


Data S1.

## Data Availability

All the data generated and described in this article are available from the corresponding web servers and are freely available to any scientist wishing to use them for noncommercial purposes without breaching participant confidentiality. All codes and R packages used in the study are publicly available and have been disclosed in the Methods section or are available from the corresponding authors upon reasonable request.
