# Supplementary material for: IGFBP7 regulates cell proliferation and migration through JAK/STAT pathway in gastric cancer and is regulated by DNA and RNA methylation
Source: J Cell Mol Med. 2024 Oct 1;28(19):e70080. doi: 10.1111/jcmm.70080 (PMC11443158; doi:10.1111/jcmm.70080)
Supplement: Supplementary file 1 — Data S1. [file JCMM-28-e70080-s001.docx]

IGFBP7 Regulates Cell Proliferation and Migration through JAK/STAT pathway in Gastric Cancer and is Regulated by DNA and RNA Methylation

Weilie Mo ^1,2^, Lijian Deng ^3,4#^, Yun Cheng ^1,2^, Sen Ge ^1,2^ and Jin Wang ^5,*^

**Supplementary Table and Figures**

| Gene | Coefficient | P value | Significant |
| --- | --- | --- | --- |
| FTO | 0.404 | < 0.001 | Positive |
| ALKBH5 | 0.140 | 0.003 | No |
| WTAP | 0.120 | 0.011 | No |
| METTL14 | 0.109 | 0.021 | No |
| YTHDF3 | 0.097 | 0.040 | No |
| YTHDC1 | 0.059 | 0.209 | No |
| IGF2BP3 | 0.036 | 0.446 | No |
| METTL3 | 0.016 | 0.728 | No |
| IGF2BP2 | 0.007 | 0.890 | No |
| YTHDF1 | 0.000 | 0.994 | No |
| IGF2BP1 | -0.002 | 0.963 | No |
| YTHDC2 | -0.014 | 0.766 | No |
| YTHDF2 | -0.017 | 0.720 | No |

**Table S1**. Correlation analysis between the expression of IGFBP7 and m^6^A related genes


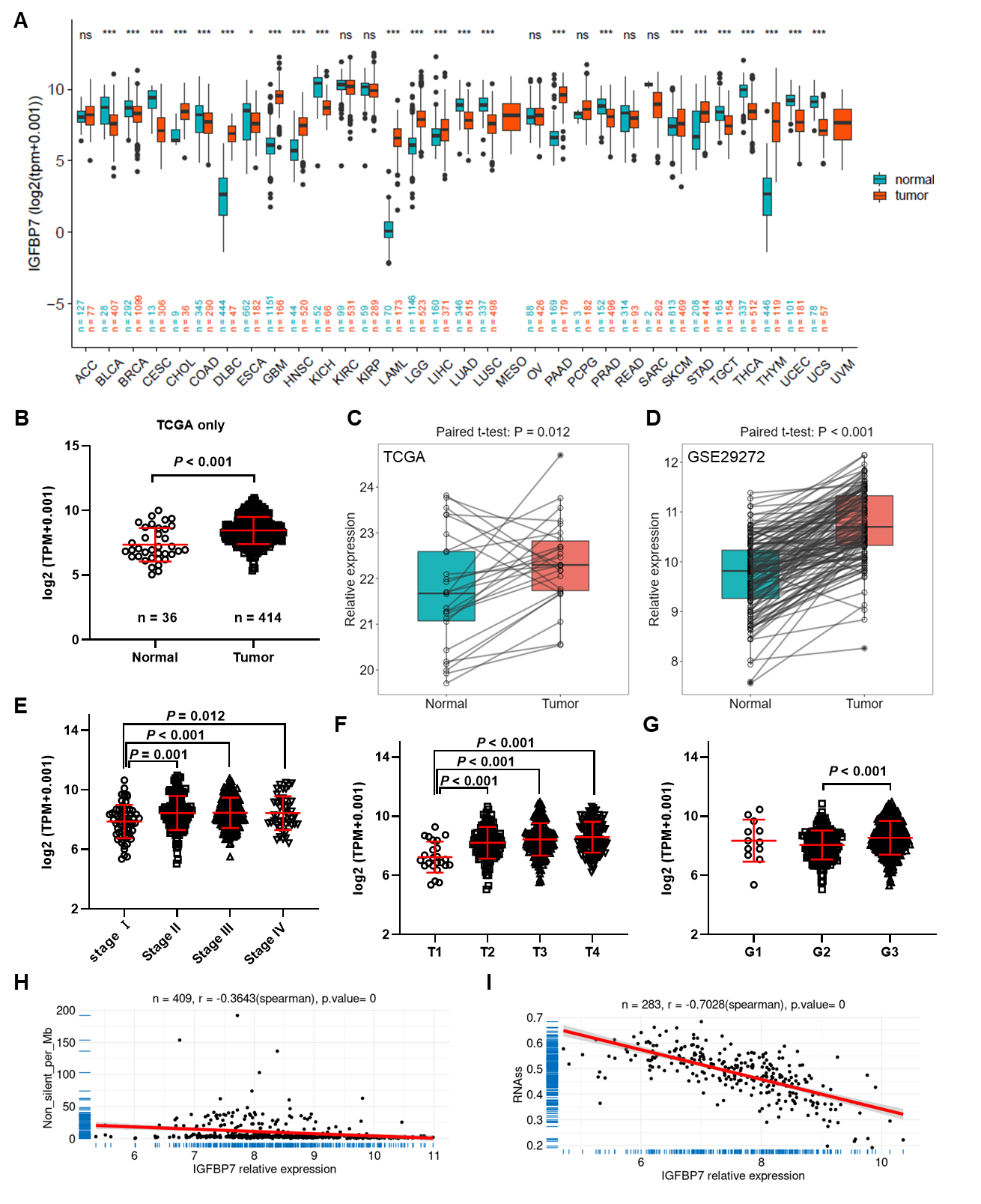


**Figure S1.** IGFBP7 was highly expressed in gastric cancer tissues and has important clinical significance. (A) Expression levels of IGFBP7 in pan-cancer based on TCGA and GTEx databases. * P < 0.05, ** P < 0.01 and *** P < 0.001 normal vs. tumor. (B) IGFBP7 expression in tumor and normal tissues of STAD samples when using TCGA database only. Boxplots show the different expression of IGFBP7 in STAD tissues compared with the paired normal tissues in TCGA-STAD (C) and GSE29272 (D) datasets. Different expression of IGFBP7 in STAD samples with different tumor stage (E), invasion depth (F) and grade (G). Scatter plot shows the correlation between IGFBP7 expression and tumor mutation burden (H) or tumor stemness (I). TCGA: The Cancer Genome Atlas. GTEx: Genotype-Tissue Expression. STAD, Stomach adenocarcinoma.


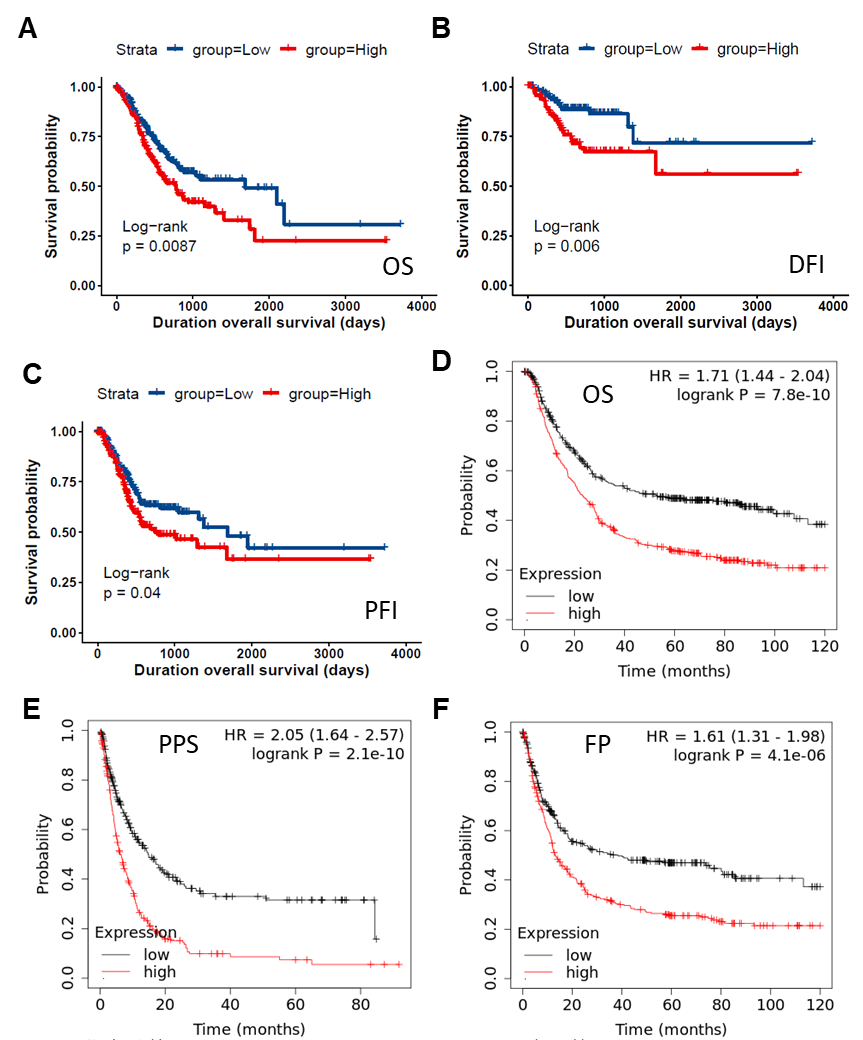


**Figure S2.** Higher IGFBP7 expression is related to poor prognosis in gastric cancer. (A-C) Survival analysis of IGFBP7 for OS, DFI and PFI based on TCGA-STAD dataset. (D-F) Survival analysis of IGFBP7 for OS, PPS and FP based on the Kaplan-Meier plotter online tool. OS, Overall survival. PFI, progression free interval. PPS, post progression survival. FP, First Progression.


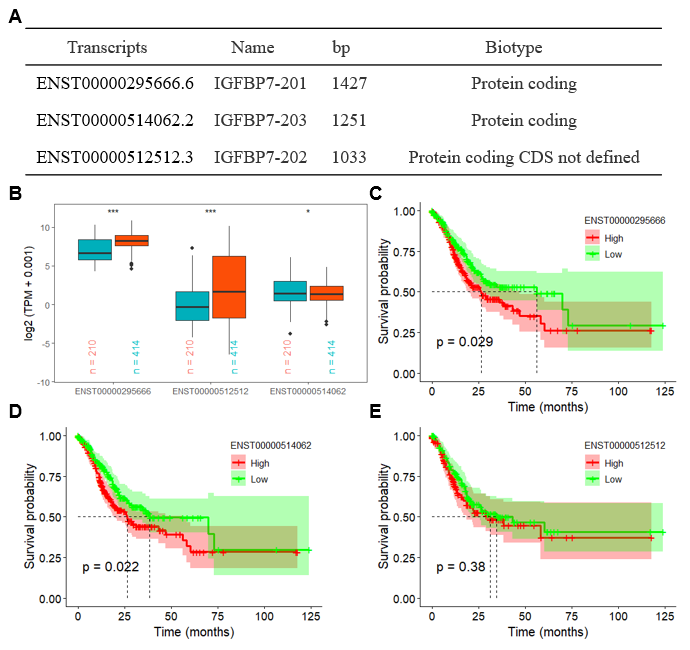


**Figure S3.** Transcripts analysis of IGFBP7. (A) Summary table of the transcripts of IGFBP7. (B) Expression analysis of the transcripts of IGFBP7 in TCGA STAD datasets. (C) Kaplan-Meier curves show the prognostic analysis of the transcripts of IGFBP for overall survival.


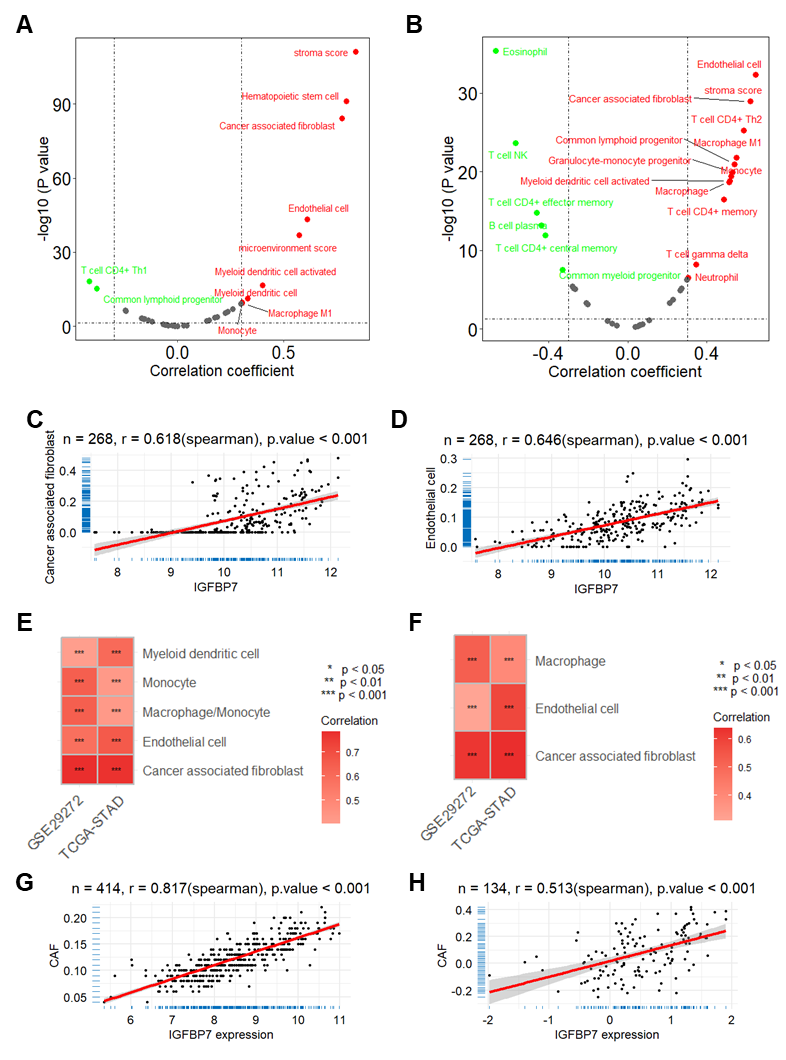


**Figure S4.** IGFBP7 correlated with immune cell infiltration. Volcano plots show the correlation between IGFBP7 expression and infiltration of immune cells calculated according to XCELL algorithm based on TCGA-STAD (A) and GSE29272 (B) datasets. Scatter plots show the correlation between IGFBP7 expression and the infiltration of cancer-associated fibroblast (C) and endothelial cell (D) in GSE29272. Heatmaps show the correlation between IGFBP7 expression and infiltration of immune cells calculated according to MCPCounter (E) and EPIC (F) algorithms based on TCGA-STAD and GSE29272 datasets. Scatter plots show the correlation between IGFBP7 expression and the infiltration of cancer-associated fibroblast in TCGA-STAD (G) and GSE29272 (H) based on TIDE online tool. TCGA: The Cancer Genome Atlas. STAD, Stomach adenocarcinoma. TIDE, Tumor Immune Dysfunction and Exclusion.


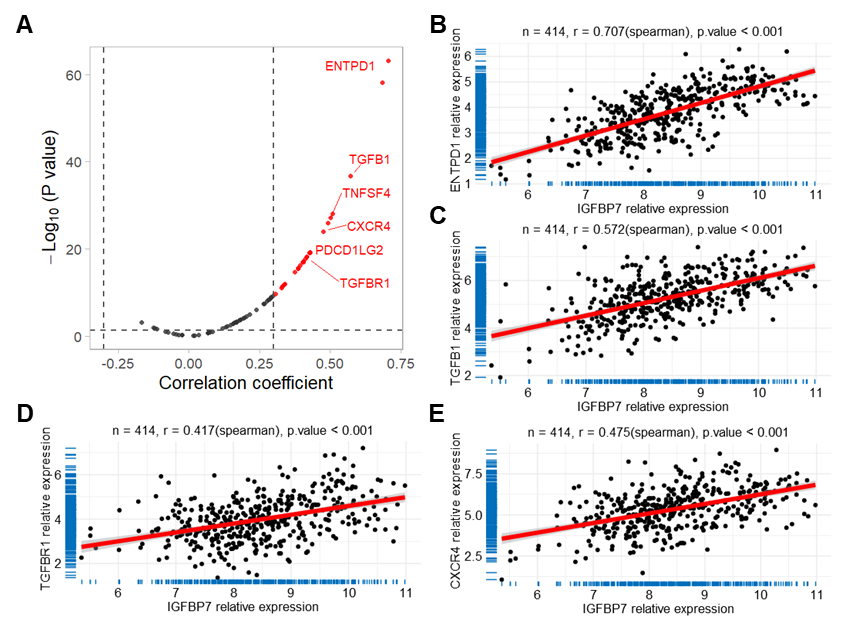


**Figure S5.** *IGFBP7* correlated with immunomodulatory molecules. (A) Volcano plot shows the results of the correlation analysis between the expression of *IGFBP7* and immunomodulatory molecules based on TCGA STAD dataset. Scatter plots show the correlation between *IGFBP7* expression and ENTPD1 (B), TGFB1 (C), TGFBR1 (D) and CXCR4 (E). TCGA: The Cancer Genome Atlas. STAD, Stomach adenocarcinoma.


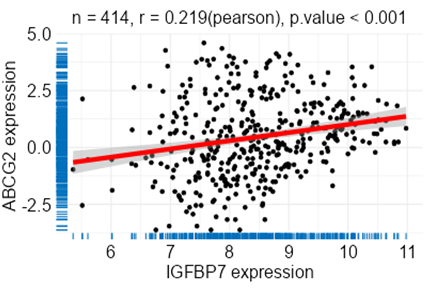


**Figure S6.** Scatter plot shows the correlation between the expression of *IGFBP7* and *ABCG2* in TCGA-BLCA dataset. TCGA: The Cancer Genome Atlas. STAD, Stomach adenocarcinoma.


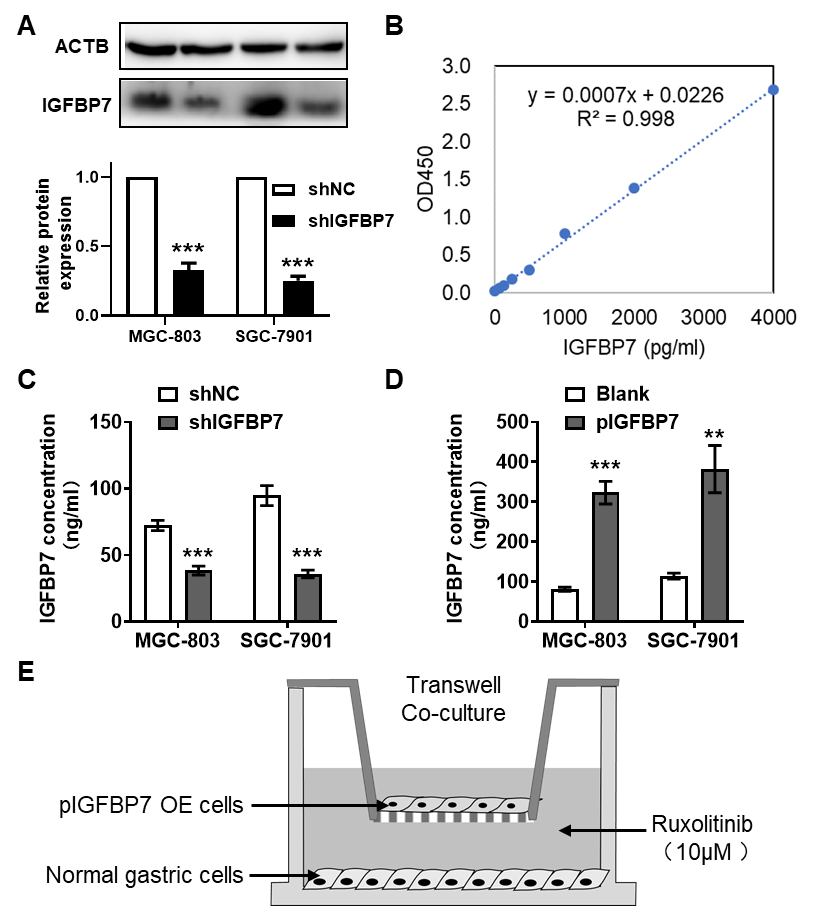


**Figure S7.** Changes in IGFBP7 expression in cell lysates and supernatants following knockdown or overexpression of IGFBP7. (A) Western blotting analysis and the statistic results of IGFBP7 proteins in GC cells transfected with shIGFBP7. (B) Standard curve of the ELISA assay for detecting IGFBP7 in cell supernatants. The IGFBP7 concentration in GC cell supernatants after knocked down (C) or overexpressed (D) IGFBP7. (E) Transwell co-culture schematic diagram. GC, gastric cancer.


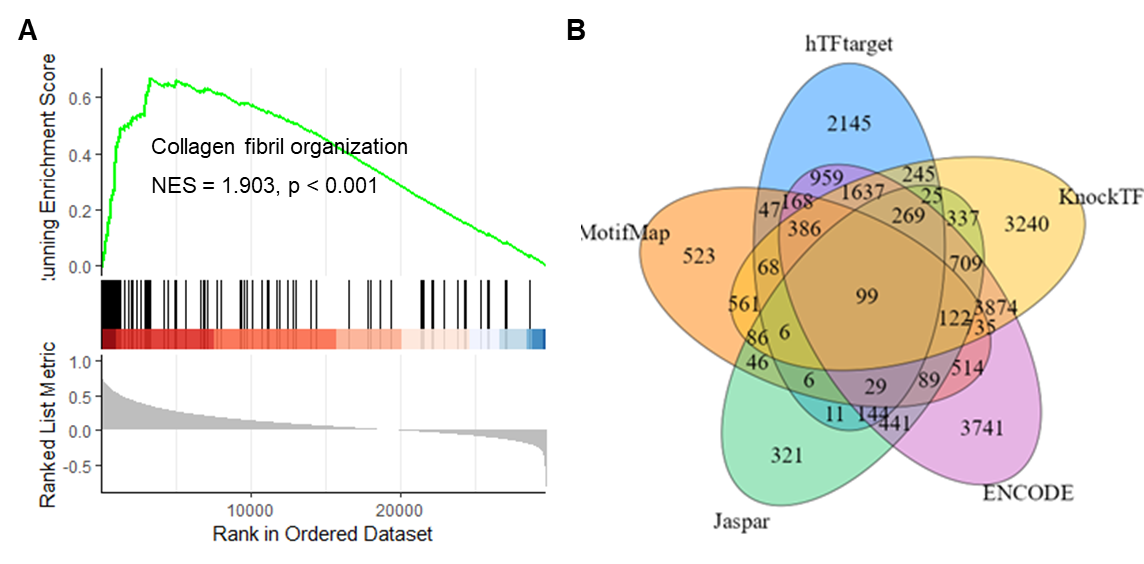


**Figure S8.** *IGFBP7* relates to JAK-STAT signaling pathway. (A) GSEA plot shows *IGFBP7* relates to JAK-STAT signaling pathway. (B) Venn diagram shows the intersection of the predicted target genes of *IGFBP7* by 5 different databases.


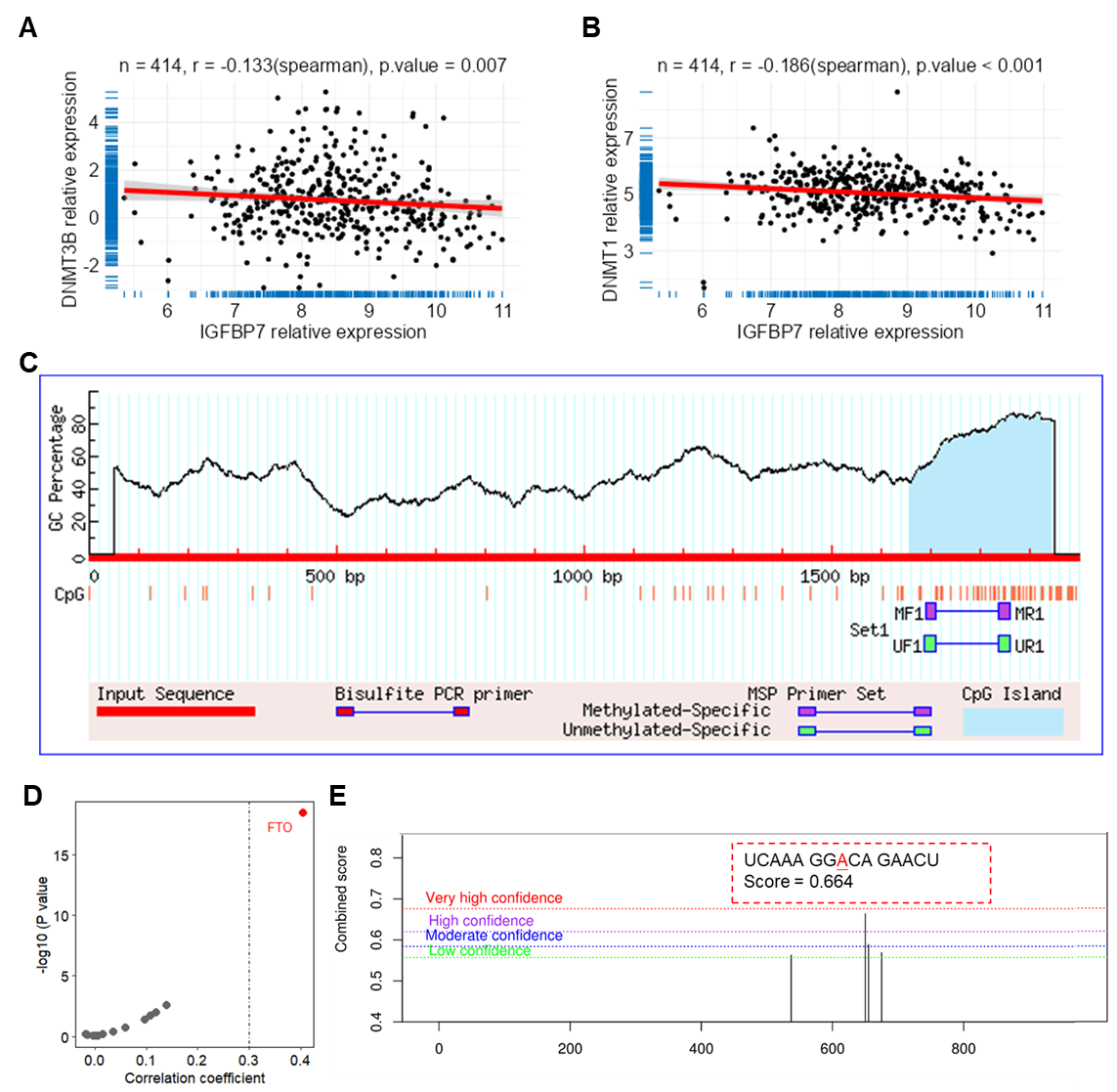


**Figure S9.** *IGFBP7* is regulated by DNA methylation. Scatter plots show the correlation between the expression between *IGFBP7* and *DNMT3B* (A) and *DNMT1* (B). CpG sites and CpGs island distribution in the promotor region of *IGFBP7*.
